# Correction: Transmembrane protease serine 2 (TMPRSS2) rs75603675, comorbidity and sex are the primary predictors of Covid-19 severity

**DOI:** 10.26508/lsa.202201545

**Published:** 2022-06-09

**Authors:** Gonzalo Villapalos-García, Pablo Zubiaur, Rebeca Rivas-Durán, Pilar Campos-Norte, Cristina Arévalo-Román, Marta Fernández-Rico, Lucio García-Fraile Fraile, Paula Fernández-Campos, Paula Soria-Chacartegui, Sara Fernández de Córdoba-Oñate, Pablo Delgado-Wicke, Elena Fernández-Ruiz, Isidoro González-Álvaro, Jesús Sanz, Francisco Abad-Santos, Ignacio de los Santos

**Affiliations:** 1 Clinical Pharmacology Department, Hospital Universitario La Princesa, Instituto Teófilo Hernando, Universidad Autónoma de Madrid (UAM), Instituto de Investigación Sanitaria La Princesa (IIS-IP), Madrid, Spain; 2 Centro de Investigación Biomédica en Red de Enfermedades Hepáticas y Digestivas (CIBERehd), Instituto de Salud Carlos III, Madrid, Spain; 3 Infectious Diseases Unit, Hospital Universitario La Princesa, Instituto de Investigación Sanitaria La Princesa (IIS-IP), Madrid, Spain; 4 Molecular Biology Unit, Hospital Universitario La Princesa, Instituto de Investigación Sanitaria La Princesa (IIS-IP), Madrid, Spain; 5 Rheumatology Service, Hospital Universitario La Princesa, Instituto de Investigación Sanitaria La Princesa (IIS-IP), Madrid, Spain

## Abstract

We identified an error in the abstract of the article: *TPMRSS2* rs75603675 OR is incorrectly indicated. It should read (OR = 2.140) instead of (OR = 0.586). We apologize for this error. However, since the main text is correct, it has no impact on the results displayed in the study.

## Abstract (precis)

We identified an error in the abstract of the article: TPMRSS2 rs75603675 OR is incorrectly indicated. It should read (OR = 2.140) instead of (OR = 0.586).

Article: Villapalos-García G, Zubiaur P, Rivas-Durán R, Campos-Norte P, Arévalo-Román C, Fernández-Rico M, García-Fraile Fraile L, Fernández-Campos P, Soria-Chacartegui P, Fernández de Córdoba-Oñate S, Delgado-Wicke P, Fernández-Ruiz E, González-Álvaro I, Sanz J, Abad-Santos F, de Los Santos I (2022 May 30) Transmembrane protease serine 2 (*TMPRSS2*) rs75603675, comorbidity, and sex are the primary predictors of COVID-19 severity. Life Sci Alliance 5(10): e202201396. doi: 10.26508/lsa.202201396. PMID: 35636966.

Where it reads:

**Abstract**. By the end of December 2021, coronavirus disease 2019 (COVID-19) produced more than 271 million cases and 5.3 million deaths. Although vaccination is an effective strategy for pandemic control, it is not yet equally available in all countries. Therefore, identification of prognostic biomarkers remains crucial to manage COVID-19 patients. The aim of this study was to evaluate predictors of COVID-19 severity previously proposed. Clinical and demographic characteristics and 120 single-nucleotide polymorphisms were analyzed from 817 patients with COVID-19, who attended the emergency department of the Hospital Universitario de La Princesa during March and April 2020. The main outcome was a modified version of the 7-point World Health Organization (WHO) COVID-19 severity scale (WHOCS); both in the moment of the first hospital examination (WHOCS-1) and of the severest WHOCS score (WHOCS-2). The *TMPRSS2* rs75603675 genotype (OR = 0.586), dyslipidemia (OR = 2.289), sex (OR = 0.586), and the Charlson Comorbidity Index (OR = 1.126) were identified as the main predictors of disease severity. Consequently, these variables might influence COVID-19 severity and could be used as predictors of disease development.

It should read:

**Abstract**. By the end of December 2021, coronavirus disease 2019 (COVID-19) produced more than 271 million cases and 5.3 million deaths. Although vaccination is an effective strategy for pandemic control, it is not yet equally available in all countries. Therefore, identification of prognostic biomarkers remains crucial to manage COVID-19 patients. The aim of this study was to evaluate predictors of COVID-19 severity previously proposed. Clinical and demographic characteristics and 120 single-nucleotide polymorphisms were analyzed from 817 patients with COVID-19, who attended the emergency department of the Hospital Universitario de La Princesa during March and April 2020. The main outcome was a modified version of the 7-point World Health Organization (WHO) COVID-19 severity scale (WHOCS); both in the moment of the first hospital examination (WHOCS-1) and of the severest WHOCS score (WHOCS-2). The *TMPRSS2* rs75603675 genotype (OR = 2.140), dyslipidemia (OR = 2.289), sex (OR = 0.586), and the Charlson Comorbidity Index (OR = 1.126) were identified as the main predictors of disease severity. Consequently, these variables might influence COVID-19 severity and could be used as predictors of disease development.

