# Supplementary material for: Transmembrane protease serine 2 (TMPRSS2) rs75603675, comorbidity, and sex are the primary predictors of COVID-19 severity
Source: Life Sci Alliance. 2022 May 30;5(10):e202201396. doi: 10.26508/lsa.202201396 (PMC9152129; doi:10.26508/lsa.202201396)
Supplement: Supplementary file 1 [file LSA-2022-01396_TableS1.docx]

**SUPPLEMENTARY MATERIAL**

**Supplementary Table 1**. Summary of nominally significant associations and whole results of the univariate analysis of A) the Severity 1 and Severity 2 variables and B) the WHOCS-1 and WHOCS-2 variables.

A)

| Summary of nominally significant results: SEVERITY 1. | | | | | |  | Summary of nominally significant results: SEVERITY 2. | | | | | |
| --- | --- | --- | --- | --- | --- | --- | --- | --- | --- | --- | --- | --- |
| Risk factor | OR | 2.5 % | 97.5 % | *p* | *p'* |  | Risk factor | OR | 2.5 % | 97.5 % | *p* | *p'* |
| HCP5 rs2395029 | 0.413 | 0.198 | 0.875 | 0.019 | 1 |  | CYP2C19 rs4244285 | 0.348 | 0.169 | 0.724 | 0.004 | 0.535 |
| HMOX1 rs2071746 | 1.577 | 1.038 | 2.376 | 0.031 | 1 |  | DPP4 rs17574 | 1.564 | 1.002 | 2.442 | 0.049 | 1.000 |
| IFNL4 rs12979860 | 2.118 | 1.325 | 3.363 | 0.002 | 0.186 |  | HCP5 rs2395029 | 0.375 | 0.184 | 0.755 | 0.006 | 0.735 |
| MTHFR rs1801133 | 0.63 | 0.403 | 0.964 | 0.037 | 1 |  | IFNL4 rs12979860 | 1.903 | 1.216 | 2.981 | 0.005 | 0.579 |
| TMPRSS2 rs75603675 | 2.1 | 1.452 | 3.032 | 7.672E-05 | 0.009 |  | NFKB1 rs28362491 | 0.658 | 0.443 | 0.973 | 0.037 | 1.000 |
| Obesity | 1.804 | 1.117 | 3.009 | 0.019 | 1 |  | TMPRSS2 rs75603675 | 1.717 | 1.212 | 2.434 | 0.002 | 0.285 |
| Dyslipemia | 2.244 | 1.527 | 3.349 | 5.287E-05 | 0.006 |  | Obesity | 1.797 | 1.213 | 2.669 | 0.004 | 0.426 |
| ACEIs | 2.471 | 1.409 | 4.561 | 0.002 | 0.293 |  | Dyslipemia | 1.849 | 1.348 | 2.546 | 0.000 | 0.018 |
| ARA-II | 1.735 | 1.047 | 2.974 | 0.038 | 1 |  | ACEIs | 1.629 | 1.073 | 2.479 | 0.022 | 1.000 |
|  |  |  |  |  |  |  | ARA-II | 1.622 | 1.082 | 2.439 | 0.019 | 1.000 |
|  |  |  |  |  |  |  |  |  |  |  |  |  |
| UNIVARIATE ANALYSIS: SEVERITY 1 | | | | | |  | UNIVARIATE ANALYSIS: SEVERITY 2 | | | | | |
| Risk factor | OR | 2.5 % | 97.5 % | *p* | *p'* |  | Risk factor | OR | 2.5 % | 97.5 % | *p* | *p'* |
| ABO rs657152 | 1.164 | 0.745 | 1.792 | 0.496 | 1.000 |  | ABO rs657152 | 1.151 | 0.777 | 1.703 | 0.483 | 1.000 |
| ACE2 rs143695310 | 0.650 | 0.281 | 1.552 | 0.318 | 1.000 |  | ACE2 rs143695310 | 0.683 | 0.308 | 1.515 | 0.345 | 1.000 |
| ACE2 rs1996225 | 0.934 | 0.659 | 1.317 | 0.697 | 1.000 |  | ACE2 rs1996225 | 0.869 | 0.639 | 1.181 | 0.372 | 1.000 |
| ACE2 rs2074192 | 1.144 | 0.798 | 1.634 | 0.461 | 1.000 |  | ACE2 rs2074192 | 1.185 | 0.863 | 1.629 | 0.294 | 1.000 |
| ACE2 rs2106809 | 1.381 | 0.851 | 2.213 | 0.185 | 1.000 |  | ACE2 rs2106809 | 1.082 | 0.693 | 1.688 | 0.728 | 1.000 |
| ACE2 rs2285666 | 0.936 | 0.580 | 1.481 | 0.782 | 1.000 |  | ACE2 rs2285666 | 0.845 | 0.559 | 1.272 | 0.420 | 1.000 |
| ACE2 rs35803318 | 1.519 | 0.680 | 3.219 | 0.288 | 1.000 |  | ACE2 rs35803318 | 1.657 | 0.81 | 3.364 | 0.163 | 1.000 |
| ACE2 rs41303171 | 0.573 | 0.066 | 3.184 | 0.570 | 1.000 |  | ACE2 rs41303171 | 0.444 | 0.097 | 2.042 | 0.295 | 1.000 |
| ACE2 rs4646156 | 0.954 | 0.638 | 1.410 | 0.816 | 1.000 |  | ACE2 rs4646156 | 0.925 | 0.654 | 1.307 | 0.661 | 1.000 |
| ACE2 rs4646188 | 1.100 | 0.511 | 2.209 | 0.796 | 1.000 |  | ACE2 rs4646188 | 1.133 | 0.592 | 2.149 | 0.703 | 1.000 |
| ACE2 rs4830542 | 1.080 | 0.721 | 1.604 | 0.706 | 1.000 |  | ACE2 rs4830542 | 1.037 | 0.726 | 1.48 | 0.841 | 1.000 |
| ACE rs1799752 | 1.966 | 0.890 | 4.260 | 0.087 | 1.000 |  | ACE rs1799752 | 1.774 | 0.829 | 3.803 | 0.138 | 1.000 |
| ACE rs4291 | 0.927 | 0.571 | 1.472 | 0.752 | 1.000 |  | ACE rs4291 | 1.03 | 0.683 | 1.55 | 0.887 | 1.000 |
| ACE rs4343 | 0.803 | 0.546 | 1.168 | 0.259 | 1.000 |  | ACE rs4343 | 0.817 | 0.585 | 1.137 | 0.232 | 1.000 |
| ADAM17 rs12692386 | 0.829 | 0.520 | 1.294 | 0.419 | 1.000 |  | ADAM17 rs12692386 | 0.945 | 0.637 | 1.4 | 0.779 | 1.000 |
| ADAM17 rs55790676 | 0.558 | 0.229 | 1.220 | 0.169 | 1.000 |  | ADAM17 rs55790676 | 0.833 | 0.448 | 1.543 | 0.564 | 1.000 |
| AGT rs699 | 0.777 | 0.529 | 1.127 | 0.189 | 1.000 |  | AGT rs699 | 0.823 | 0.592 | 1.143 | 0.247 | 1.000 |
| APOE rs429358 | 1.853 | 0.545 | 5.918 | 0.299 | 1.000 |  | APOE rs429358 | 2.156 | 0.714 | 6.595 | 0.169 | 1.000 |
| APOE rs7412 | 0.180 | 0.015 | 1.958 | 0.206 | 1.000 |  | APOE rs7412 | 0.693 | 0.135 | 3.575 | 0.659 | 1.000 |
| BSG rs8259 | 0.889 | 0.522 | 1.470 | 0.653 | 1.000 |  | BSG rs8259 | 0.798 | 0.502 | 1.264 | 0.339 | 1.000 |
| CCL2 rs1024611 | 0.578 | 0.327 | 0.983 | 0.050 | 1.000 |  | CCL2 rs1024611 | 0.648 | 0.415 | 1.007 | 0.055 | 1.000 |
| CCL5 rs2107538 | 0.433 | 0.162 | 1.003 | 0.069 | 1.000 |  | CCL5 rs2107538 | 0.58 | 0.296 | 1.13 | 0.111 | 1.000 |
| CD14 rs2569190 | 1.105 | 0.673 | 1.806 | 0.691 | 1.000 |  | CD14 rs2569190 | 1.172 | 0.756 | 1.824 | 0.481 | 1.000 |
| CD69 rs11052877 | 0.866 | 0.527 | 1.387 | 0.560 | 1.000 |  | CD69 rs11052877 | 0.965 | 0.634 | 1.462 | 0.867 | 1.000 |
| CLEC2D rs1560011 | 1.205 | 0.769 | 1.857 | 0.406 | 1.000 |  | CLEC2D rs1560011 | 1.234 | 0.835 | 1.822 | 0.291 | 1.000 |
| CRP rs1130864 | 1.332 | 0.817 | 2.136 | 0.241 | 1.000 |  | CRP rs1130864 | 1.264 | 0.81 | 1.969 | 0.301 | 1.000 |
| CSF3 rs2227322 | 1.067 | 0.660 | 1.692 | 0.785 | 1.000 |  | CSF3 rs2227322 | 1.034 | 0.678 | 1.572 | 0.876 | 1.000 |
| CXCL1 rs2071425 | 0.654 | 0.350 | 1.165 | 0.165 | 1.000 |  | CXCL1 rs2071425 | 0.839 | 0.512 | 1.369 | 0.485 | 1.000 |
| CYP2C19 rs12248560 | 1.555 | 0.660 | 3.437 | 0.289 | 1.000 |  | CYP2C19 rs12248560 | 1.751 | 0.821 | 3.692 | 0.143 | 1.000 |
| CYP2C19 rs4244285 | 0.958 | 0.416 | 2.028 | 0.914 | 1.000 |  | CYP2C19 rs4244285 | 0.348 | 0.169 | 0.724 | 0.004 | 0.535 |
| CYP2C9 rs1057910 | 0.110 | 0.002 | 5.035 | 0.395 | 1.000 |  | CYP2C9 rs1057910 | 0.082 | 0.003 | 1.368 | 0.071 | 1.000 |
| CYP2C9 rs1799853 | 1.065 | 0.340 | 2.939 | 0.907 | 1.000 |  | CYP2C9 rs1799853 | 0.853 | 0.309 | 2.313 | 0.756 | 1.000 |
| CYP3A4 rs35599367 | 0.182 | 0.003 | 11.077 | 0.452 | 1.000 |  | CYP3A4 rs35599367 | 0.788 | 0.028 | 21.715 | 0.873 | 1.000 |
| CYP3A4 rs67666821 | 0.336 | 0.049 | 2.657 | 0.261 | 1.000 |  | CYP3A4 rs67666821 | 0.278 | 0.044 | 1.591 | 0.151 | 1.000 |
| CYP3A5 rs776746 | 1.335 | 0.410 | 3.900 | 0.608 | 1.000 |  | CYP3A5 rs776746 | 1.625 | 0.594 | 4.396 | 0.338 | 1.000 |
| CYP4V2 rs13146272 | 0.772 | 0.515 | 1.142 | 0.203 | 1.000 |  | CYP4V2 rs13146272 | 0.801 | 0.568 | 1.128 | 0.205 | 1.000 |
| DPP4 rs116302758 | 2.347 | 0.093 | 62.279 | 0.556 | 1.000 |  | DPP4 rs116302758 | 2.666 | 0.162 | 68.749 | 0.484 | 1.000 |
| DPP4 rs17574 | 1.415 | 0.854 | 2.301 | 0.169 | 1.000 |  | DPP4 rs17574 | 1.564 | 1.002 | 2.442 | 0.049 | 1.000 |
| DPP4 rs56179129 | 0.547 | 0.178 | 1.841 | 0.300 | 1.000 |  | DPP4 rs56179129 | 0.778 | 0.246 | 2.545 | 0.672 | 1.000 |
| ENOX1 rs9594987 | 0.989 | 0.677 | 1.431 | 0.955 | 1.000 |  | ENOX1 rs9594987 | 0.892 | 0.637 | 1.247 | 0.504 | 1.000 |
| EPHX1 rs1051740 | 0.652 | 0.362 | 1.120 | 0.136 | 1.000 |  | EPHX1 rs1051740 | 0.669 | 0.421 | 1.059 | 0.088 | 1.000 |
| F11 rs2036914 | 0.983 | 0.666 | 1.437 | 0.932 | 1.000 |  | F11 rs2036914 | 1.102 | 0.788 | 1.539 | 0.570 | 1.000 |
| F11 rs2289252 | 0.883 | 0.557 | 1.372 | 0.586 | 1.000 |  | F11 rs2289252 | 0.846 | 0.57 | 1.252 | 0.404 | 1.000 |
| FGG rs2066865 | 0.726 | 0.292 | 1.627 | 0.461 | 1.000 |  | FGG rs2066865 | 0.679 | 0.32 | 1.421 | 0.308 | 1.000 |
| G6PD rs1050828 | 1.432 | 0.184 | 21.967 | 0.752 | 1.000 |  | G6PD rs1050828 | 1.172 | 0.176 | 8.442 | 0.870 | 1.000 |
| G6PD rs1050829 | 1.778 | 0.268 | 22.451 | 0.594 | 1.000 |  | G6PD rs1050829 | 1.328 | 0.247 | 7.638 | 0.744 | 1.000 |
| GC rs4588 | 0.821 | 0.416 | 1.529 | 0.549 | 1.000 |  | GC rs4588 | 0.929 | 0.539 | 1.593 | 0.788 | 1.000 |
| GC rs7041 | 0.842 | 0.560 | 1.248 | 0.400 | 1.000 |  | GC rs7041 | 0.899 | 0.635 | 1.272 | 0.549 | 1.000 |
| HCP5 rs2395029 | 0.413 | 0.198 | 0.875 | 0.019 | 1.000 |  | HCP5 rs2395029 | 0.375 | 0.184 | 0.755 | 0.006 | 0.735 |
| HMOX1 rs2071746 | 1.577 | 1.038 | 2.376 | 0.031 | 1.000 |  | HMOX1 rs2071746 | 1.371 | 0.932 | 2.016 | 0.108 | 1.000 |
| IFITM3 rs12252 | 3.072 | 0.407 | 18.835 | 0.222 | 1.000 |  | IFITM3 rs12252 | 3.238 | 0.511 | 19.345 | 0.187 | 1.000 |
| IFNL4 rs12979860 | 2.118 | 1.325 | 3.363 | 0.002 | 0.186 |  | IFNL4 rs12979860 | 1.903 | 1.216 | 2.981 | 0.005 | 0.579 |
| IL10 rs1800871 | 0.770 | 0.428 | 1.329 | 0.365 | 1.000 |  | IL10 rs1800871 | 0.93 | 0.578 | 1.491 | 0.765 | 1.000 |
| IL10 rs1800896 | 1.333 | 0.857 | 2.048 | 0.195 | 1.000 |  | IL10 rs1800896 | 1.339 | 0.901 | 1.988 | 0.149 | 1.000 |
| IL13 rs1800925 | 1.456 | 0.592 | 3.343 | 0.390 | 1.000 |  | IL13 rs1800925 | 1.181 | 0.525 | 2.634 | 0.685 | 1.000 |
| IL17A rs2275913 | 1.154 | 0.653 | 1.978 | 0.611 | 1.000 |  | IL17A rs2275913 | 1.194 | 0.726 | 1.958 | 0.484 | 1.000 |
| IL17A rs3819025 | 0.572 | 0.228 | 1.291 | 0.203 | 1.000 |  | IL17A rs3819025 | 0.726 | 0.364 | 1.437 | 0.360 | 1.000 |
| IL1B rs1143627 | 0.946 | 0.603 | 1.458 | 0.805 | 1.000 |  | IL1B rs1143627 | 0.886 | 0.599 | 1.308 | 0.544 | 1.000 |
| IL1B rs1143634 | 0.759 | 0.314 | 1.677 | 0.516 | 1.000 |  | IL1B rs1143634 | 0.71 | 0.351 | 1.428 | 0.339 | 1.000 |
| IL1RN rs315952 | 1.356 | 0.647 | 2.727 | 0.404 | 1.000 |  | IL1RN rs315952 | 1.14 | 0.578 | 2.233 | 0.704 | 1.000 |
| IL6R rs11265618 | 1.681 | 0.734 | 3.697 | 0.204 | 1.000 |  | IL6R rs11265618 | 1.729 | 0.81 | 3.679 | 0.155 | 1.000 |
| IL6R rs12083537 | 0.903 | 0.409 | 1.861 | 0.791 | 1.000 |  | IL6R rs12083537 | 1.061 | 0.552 | 2.025 | 0.858 | 1.000 |
| IL6R rs2228145 | 0.773 | 0.475 | 1.225 | 0.284 | 1.000 |  | IL6R rs2228145 | 0.831 | 0.554 | 1.243 | 0.370 | 1.000 |
| IL6R rs4329505 | 1.521 | 0.605 | 3.574 | 0.349 | 1.000 |  | IL6R rs4329505 | 1.913 | 0.86 | 4.232 | 0.109 | 1.000 |
| IL6R rs7529229 | 0.712 | 0.447 | 1.109 | 0.142 | 1.000 |  | IL6R rs7529229 | 0.744 | 0.507 | 1.09 | 0.130 | 1.000 |
| IL6 rs1800795 | 2.116 | 0.089 | 53.968 | 0.596 | 1.000 |  | IL6 rs1800795 | 2.12 | 0.129 | 52.065 | 0.583 | 1.000 |
| IL6 rs1800796 | 1.533 | 0.921 | 2.508 | 0.093 | 1.000 |  | IL6 rs1800796 | 1.318 | 0.821 | 2.113 | 0.251 | 1.000 |
| IL6 rs1818879 | 0.452 | 0.173 | 1.044 | 0.082 | 1.000 |  | IL6 rs1818879 | 0.502 | 0.249 | 1.009 | 0.054 | 1.000 |
| KCNIP1 rs703505 | 0.950 | 0.625 | 1.424 | 0.808 | 1.000 |  | KCNIP1 rs703505 | 0.903 | 0.627 | 1.299 | 0.583 | 1.000 |
| LZTFL1 rs35044562 | 0.219 | 0.009 | 4.987 | 0.435 | 1.000 |  | LZTFL1 rs35044562 | 1.003 | 0.12 | 8.382 | 0.998 | 1.000 |
| MTHFR rs1801131 | 0.677 | 0.346 | 1.252 | 0.233 | 1.000 |  | MTHFR rs1801131 | 0.639 | 0.376 | 1.085 | 0.098 | 1.000 |
| MTHFR rs1801133 | 0.630 | 0.403 | 0.964 | 0.037 | 1.000 |  | MTHFR rs1801133 | 0.769 | 0.537 | 1.098 | 0.149 | 1.000 |
| MX1 rs469390 | 0.736 | 0.492 | 1.085 | 0.128 | 1.000 |  | MX1 rs469390 | 0.817 | 0.584 | 1.141 | 0.236 | 1.000 |
| NFKB1 rs28362491 | 0.840 | 0.537 | 1.291 | 0.436 | 1.000 |  | NFKB1 rs28362491 | 0.658 | 0.443 | 0.973 | 0.037 | 1.000 |
| NLRP3 rs10754555 | 0.866 | 0.521 | 1.399 | 0.567 | 1.000 |  | NLRP3 rs10754555 | 0.782 | 0.505 | 1.205 | 0.267 | 1.000 |
| PEAR1 rs12041331 | 0.378 | 0.075 | 1.360 | 0.180 | 1.000 |  | PEAR1 rs12041331 | 0.565 | 0.198 | 1.581 | 0.280 | 1.000 |
| rs10108210 | 1.125 | 0.723 | 1.722 | 0.594 | 1.000 |  | rs10108210 | 1.1 | 0.751 | 1.61 | 0.623 | 1.000 |
| rs703297 | 0.851 | 0.572 | 1.249 | 0.417 | 1.000 |  | rs703297 | 0.882 | 0.628 | 1.238 | 0.470 | 1.000 |
| rs713400 | 0.400 | 0.054 | 1.900 | 0.316 | 1.000 |  | rs713400 | 0.628 | 0.183 | 2.148 | 0.461 | 1.000 |
| rs8134378 | 0.240 | 0.011 | 4.433 | 0.402 | 1.000 |  | rs8134378 | 0.807 | 0.094 | 6.734 | 0.842 | 1.000 |
| SLCO1B1 rs4149056 | 0.947 | 0.372 | 2.203 | 0.903 | 1.000 |  | SLCO1B1 rs4149056 | 0.542 | 0.235 | 1.24 | 0.148 | 1.000 |
| TLR1 rs5743551 | 1.166 | 0.753 | 1.778 | 0.483 | 1.000 |  | TLR1 rs5743551 | 1.088 | 0.738 | 1.6 | 0.670 | 1.000 |
| TLR2 rs11938228 | 1.307 | 0.720 | 2.285 | 0.362 | 1.000 |  | TLR2 rs11938228 | 1.406 | 0.847 | 2.321 | 0.185 | 1.000 |
| TLR2 rs1816702 | 0.941 | 0.219 | 3.227 | 0.928 | 1.000 |  | TLR2 rs1816702 | 1.072 | 0.341 | 3.313 | 0.905 | 1.000 |
| TLR2 rs1898830 | 1.022 | 0.635 | 1.611 | 0.927 | 1.000 |  | TLR2 rs1898830 | 1.13 | 0.745 | 1.709 | 0.562 | 1.000 |
| TLR2 rs3804099 | 0.961 | 0.636 | 1.433 | 0.848 | 1.000 |  | TLR2 rs3804099 | 0.902 | 0.631 | 1.287 | 0.570 | 1.000 |
| TLR2 rs7656411 | 0.914 | 0.501 | 1.596 | 0.761 | 1.000 |  | TLR2 rs7656411 | 0.811 | 0.485 | 1.345 | 0.420 | 1.000 |
| TLR4 rs1927911 | 0.854 | 0.465 | 1.504 | 0.596 | 1.000 |  | TLR4 rs1927911 | 1.053 | 0.637 | 1.731 | 0.840 | 1.000 |
| TLR4 rs5030728 | 0.871 | 0.461 | 1.573 | 0.657 | 1.000 |  | TLR4 rs5030728 | 0.765 | 0.448 | 1.302 | 0.325 | 1.000 |
| TLR9 rs187084 | 0.824 | 0.520 | 1.279 | 0.399 | 1.000 |  | TLR9 rs187084 | 0.987 | 0.673 | 1.443 | 0.946 | 1.000 |
| TLR9 rs352162 | 0.784 | 0.527 | 1.151 | 0.222 | 1.000 |  | TLR9 rs352162 | 0.851 | 0.607 | 1.191 | 0.348 | 1.000 |
| TMPRSS2 rs12329760 | 1.211 | 0.469 | 2.862 | 0.674 | 1.000 |  | TMPRSS2 rs12329760 | 1.288 | 0.578 | 2.844 | 0.533 | 1.000 |
| TMPRSS2 rs2070788 | 1.215 | 0.823 | 1.775 | 0.321 | 1.000 |  | TMPRSS2 rs2070788 | 1.211 | 0.857 | 1.71 | 0.278 | 1.000 |
| TMPRSS2 rs463727 | 1.124 | 0.716 | 1.736 | 0.605 | 1.000 |  | TMPRSS2 rs463727 | 1.079 | 0.727 | 1.599 | 0.705 | 1.000 |
| TMPRSS2 rs464397 | 1.255 | 0.833 | 1.871 | 0.270 | 1.000 |  | TMPRSS2 rs464397 | 1.269 | 0.88 | 1.828 | 0.201 | 1.000 |
| TMPRSS2 rs55964536 | 0.725 | 0.500 | 1.043 | 0.086 | 1.000 |  | TMPRSS2 rs55964536 | 0.84 | 0.613 | 1.149 | 0.276 | 1.000 |
| TMPRSS2 rs734056 | 1.082 | 0.729 | 1.588 | 0.693 | 1.000 |  | TMPRSS2 rs734056 | 1.051 | 0.743 | 1.485 | 0.777 | 1.000 |
| TMPRSS2 rs75603675 | 2.100 | 1.452 | 3.032 | 0.000 | 0.009 |  | TMPRSS2 rs75603675 | 1.717 | 1.212 | 2.434 | 0.002 | 0.285 |
| TMPRSS2 rs77675406 | 0.490 | 0.054 | 2.597 | 0.465 | 1.000 |  | TMPRSS2 rs77675406 | 0.639 | 0.16 | 2.539 | 0.527 | 1.000 |
| TNF rs1799964 | 1.111 | 0.541 | 2.173 | 0.764 | 1.000 |  | TNF rs1799964 | 1.257 | 0.68 | 2.31 | 0.463 | 1.000 |
| TNF rs1800610 | 0.912 | 0.379 | 2.016 | 0.828 | 1.000 |  | TNF rs1800610 | 0.935 | 0.46 | 1.885 | 0.852 | 1.000 |
| TNF rs1800629 | 1.910 | 0.482 | 6.927 | 0.328 | 1.000 |  | TNF rs1800629 | 1.272 | 0.333 | 4.806 | 0.722 | 1.000 |
| TNF rs361525 | 2.373 | 0.434 | 13.190 | 0.298 | 1.000 |  | TNF rs361525 | 2.672 | 0.55 | 14.469 | 0.222 | 1.000 |
| TNKA rs1800630 | 1.261 | 0.670 | 2.298 | 0.459 | 1.000 |  | TNKA rs1800630 | 1.347 | 0.772 | 2.342 | 0.292 | 1.000 |
| TRAF3IP2 rs13190932 | 0.860 | 0.073 | 5.838 | 0.889 | 1.000 |  | TRAF3IP2 rs13190932 | 1.345 | 0.249 | 7.166 | 0.727 | 1.000 |
| TRAF3IP2 rs13196377 | 0.667 | 0.066 | 4.028 | 0.694 | 1.000 |  | TRAF3IP2 rs13196377 | 1.12 | 0.24 | 5.109 | 0.884 | 1.000 |
| TRAF3IP2 rs33980500 | 0.854 | 0.073 | 5.784 | 0.884 | 1.000 |  | TRAF3IP2 rs33980500 | 1.348 | 0.25 | 7.158 | 0.725 | 1.000 |
| VDR rs2228570 | 1.032 | 0.667 | 1.573 | 0.884 | 1.000 |  | VDR rs2228570 | 1.234 | 0.848 | 1.793 | 0.271 | 1.000 |
| ABCB1 rs1045642 | 1.306 | 0.908 | 1.865 | 0.146 | 1.000 |  | ABCB1 rs1045642 | 1.32 | 0.952 | 1.83 | 0.096 | 1.000 |
| ABCB1 rs1128503 | 1.087 | 0.721 | 1.620 | 0.685 | 1.000 |  | ABCB1 rs1128503 | 1.162 | 0.811 | 1.664 | 0.413 | 1.000 |
| Obesity | 1.804 | 1.117 | 3.009 | 0.019 | 1.000 |  | Obesity | 1.797 | 1.213 | 2.669 | 0.004 | 0.426 |
| Dyslipemia | 2.244 | 1.527 | 3.349 | 0.000 | 0.006 |  | Dyslipemia | 1.849 | 1.348 | 2.546 | 0.000 | 0.018 |
| Tobacco | 1.108 | 0.760 | 1.630 | 0.599 | 1.000 |  | Tobacco | 1.186 | 0.853 | 1.651 | 0.311 | 1.000 |
| Alcohol | 1.221 | 0.665 | 2.349 | 0.534 | 1.000 |  | Alcohol | 1.117 | 0.672 | 1.858 | 0.669 | 1.000 |
| ACEi | 2.471 | 1.409 | 4.561 | 0.002 | 0.293 |  | ACEi | 1.629 | 1.073 | 2.479 | 0.022 | 1.000 |
| ARA-II | 1.735 | 1.047 | 2.974 | 0.038 | 1.000 |  | ARA-II | 1.622 | 1.082 | 2.439 | 0.019 | 1.000 |
| Antialdosterone drugs | 3.328 | 0.448 | 26.891 | 0.283 | 1.000 |  | Antialdosterone drugs | 1.468 | 0.352 | 6.036 | 0.596 | 1.000 |
| Anticoagulant drugs | 1.946 | 0.724 | 5.887 | 0.215 | 1.000 |  | Anticoagulant drugs | 1.575 | 0.76 | 3.244 | 0.220 | 1.000 |
| Corticosteroids | 3.097 | 0.960 | 11.563 | 0.079 | 1.000 |  | Corticosteroids | 1.536 | 0.67 | 3.531 | 0.312 | 1.000 |
| Immunosuppressants | 1.313 | 0.493 | 4.067 | 0.609 | 1.000 |  | Immunosuppressants | 1.084 | 0.463 | 2.566 | 0.853 | 1.000 |
| Biogeographic group | 1.414 | 0.953 | 2.097 | 0.091 | 1.000 |  | Biogeographic group | 1.253 | 0.892 | 1.763 | 0.194 | 1.000 |

**B)**

| Summary of nominally significant results: WHOCS-1 | | | | |  | Summary of nominally significant results: WHOCS-2 | | | | |
| --- | --- | --- | --- | --- | --- | --- | --- | --- | --- | --- |
|  | Estimate | SE | P | p' |  |  | Estimate | SE | p | p' |
| (Intercept) | 2.042 | 0.157 | <0.001 | <0.001 |  | (Intercept) | 2.674 | 0.142 | <0.001 | <0.001 |
| *TMPRSS2* rs75603675 | 0.600 | 0.140 | <0.001 | 0.002 |  | *TMPRSS2* rs75603675 | 0.414 | 0.126 | 0.001 | 0.135 |
| Sex | -0.329 | 0.124 | 0.008 | 0.969 |  | Sex | -0.419 | 0.112 | <0.001 | 0.024 |
| CCI | 0.143 | 0.029 | <0.001 | <0.001 |  | CCI | 0.159 | 0.026 | <0.001 | <0.001 |
|  | Estimate | SE | P | p' |  |  | Estimate | SE | p | p' |
| (Intercept) | 2.412 | 0.108 | <0.001 | <0.001 |  | (Intercept) | 2.952 | 0.101 | <0.001 | <0.001 |
| Dyslipidemia | 0.564 | 0.131 | <0.001 | 0.002 |  | Dyslipidemia | 0.467 | 0.122 | <0.001 | 0.017 |
| Sex | -0.338 | 0.117 | 0.004 | 0.504 |  | Sex | -0.449 | 0.110 | <0.001 | 0.006 |
| CCI | 0.107 | 0.028 | <0.001 | 0.023 |  | CCI | 0.142 | 0.027 | <0.001 | <0.001 |
|  | Estimate | SE | P | p' |  |  | Estimate | SE | p | p' |
| (Intercept) | 2.087 | 0.199 | <0.001 | <0.001 |  | (Intercept) | 2.667 | 0.183 | <0.001 | <0.001 |
| *IFNL4* rs12979860 | 0.465 | 0.187 | 0.013 | 1.000 |  | *IFNL4* rs12979860 | 0.413 | 0.172 | 0.017 | 1.000 |
| Sex | -0.353 | 0.120 | 0.003 | 0.397 |  | Sex | -0.476 | 0.110 | <0.001 | 0.002 |
| CCI | 0.144 | 0.028 | <0.001 | <0.001 |  | CCI | 0.161 | 0.026 | <0.001 | <0.001 |
|  | Estimate | SE | P | p' |  |  | Estimate | SE | p | p' |
| (Intercept) | 2.447 | 0.109 | <0.001 | <0.001 |  | (Intercept) | 2.961 | 0.102 | <0.001 | <0.001 |
| Obesity | 0.369 | 0.163 | 0.024 | 1.000 |  | Obesity | 0.445 | 0.152 | 0.003 | 1.000 |
| Sex | -0.316 | 0.119 | 0.008 | 0.965 |  | Sex | -0.424 | 0.110 | <0.001 | 0.016 |
| CCI | 0.140 | 0.027 | <0.001 | <0.001 |  | CCI | 0.167 | 0.025 | <0.001 | <0.001 |
|  | Estimate | SE | P | p' |  |  | Estimate | SE | p | p' |
| (Intercept) | 2.461 | 0.107 | <0.001 | <0.001 |  | (Intercept) | 2.995 | 0.100 | <0.001 | <0.001 |
| ACEIs | 0.571 | 0.176 | 0.001 | 1.000 |  | ACEIs | 0.435 | 0.165 | 0.008 | 1.000 |
| Sex | -0.328 | 0.118 | 0.006 | 0.679 |  | Sex | -0.441 | 0.110 | <0.001 | 0.008 |
| CCI | 0.129 | 0.028 | <0.001 | <0.001 |  | CCI | 0.162 | 0.026 | <0.001 | <0.001 |
|  | Estimate | SE | P | p' |  |  | Estimate | SE | p | p' |
| (Intercept) | 2.478 | 0.107 | <0.001 | <0.001 |  | (Intercept) | 3.004 | 0.100 | <0.001 | <0.001 |
| ARA-II | 0.390 | 0.170 | 0.022 | 1.000 |  | ARA-II | 0.377 | 0.159 | 0.018 | 1.000 |
| Sex | -0.327 | 0.118 | 0.006 | 0.716 |  | Sex | -0.439 | 0.110 | <0.001 | 0.009 |
| CCI | 0.129 | 0.028 | <0.001 | 0.001 |  | CCI | 0.158 | 0.026 | <0.001 | <0.001 |
|  | Estimate | SE | P | p' |  |  | Estimate | SE | p | p' |
| (Intercept) | 2.565 | 0.110 | <0.001 | <0.001 |  | (Intercept) | 3.090 | 0.101 | <0.001 | <0.001 |
| *HCP5* rs2395029 | -0.650 | 0.303 | 0.032 | 1.000 |  | *HCP5* rs2395029 | -0.674 | 0.279 | 0.016 | 1.000 |
| Sex | -0.383 | 0.119 | 0.001 | 0.170 |  | Sex | -0.485 | 0.110 | <0.001 | 0.001 |
| CCI | 0.138 | 0.028 | <0.001 | <0.001 |  | CCI | 0.154 | 0.026 | <0.001 | <0.001 |
|  | Estimate | SE | P | p' |  |  | Estimate | SE | p | p' |
| (Intercept) | 1.864 | 0.321 | <0.001 | <0.001 |  | (Intercept) | 2.628 | 0.177 | <0.001 | <0.001 |
| *ACE* rs1799752 | 0.627 | 0.319 | 0.050 | 1.000 |  | *DPP4* rs17574 | 0.481 | 0.171 | 0.005 | 1.000 |
| Sex | -0.325 | 0.120 | 0.007 | 0.819 |  | Sex | -0.486 | 0.110 | <0.001 | 0.001 |
| CCI | 0.156 | 0.028 | <0.001 | <0.001 |  | CCI | 0.162 | 0.025 | <0.001 | <0.001 |
|  | Estimate | SE | P | p' |  |  | Estimate | SE | p | p' |
| (Intercept) | 2.171 | 0.176 | <0.001 | <0.001 |  | (Intercept) | 2.756 | 0.165 | <0.001 | <0.001 |
| *HMOX1* rs2071746 | 0.374 | 0.160 | 0.020 | 1.000 |  | *IL10* rs1800896 | 0.314 | 0.152 | 0.039 | 1.000 |
| Sex | -0.322 | 0.119 | 0.007 | 0.853 |  | Sex | -0.467 | 0.110 | <0.001 | 0.003 |
| CCI | 0.150 | 0.028 | <0.001 | <0.001 |  | CCI | 0.166 | 0.026 | <0.001 | <0.001 |
|  | Estimate | SE | P | p' |  |  | Estimate | SE | p | p' |
| (Intercept) | 3.068 | 0.303 | <0.001 | <0.001 |  | (Intercept) | 3.337 | 0.157 | <0.001 | <0.001 |
| *IL6* rs1818879 | -0.592 | 0.296 | 0.046 | 1.000 |  | *NFKB1* rs28362491 | -0.360 | 0.145 | 0.013 | 1.000 |
| Sex | -0.347 | 0.119 | 0.004 | 0.450 |  | Sex | -0.473 | 0.111 | <0.001 | 0.003 |
| CCI | 0.148 | 0.028 | <0.001 | <0.001 |  | CCI | 0.156 | 0.026 | <0.001 | <0.001 |
|  | Estimate | SE | P | p' |  |  |  |  |  |  |
| (Intercept) | 2.756 | 0.156 | <0.001 | <0.001 |  |  |  |  |  |  |
| *MX1* rs469390 | -0.311 | 0.141 | 0.027 | 1.000 |  |  |  |  |  |  |
| Sex | -0.348 | 0.119 | 0.004 | 0.435 |  |  |  |  |  |  |
| CCI | 0.139 | 0.028 | <0.001 | <0.001 |  |  |  |  |  |  |
|  |  |  |  |  |  |  |  |  |  |  |
|  | UNIVARIATE ANALYSIS: WHOCS-1 | | | |  | UNIVARIATE ANALYSIS: WHOCS-2 | | | |  |
|  |  | Estimate | SE | p |  |  | Estimate | SE | p |  |
|  | (Intercept) | 3.190 | 0.092 | 0.000 |  | (Intercept) | 3.406 | 0.124 | 0.000 |  |
|  | ABO rs657152 | 0.019 | 0.085 | 0.819 |  | ABO rs657152 | 0.043 | 0.115 | 0.706 |  |
|  | Sex | -0.209 | 0.062 | 0.001 |  | Sex | -0.377 | 0.083 | 0.000 |  |
|  | CCI | 0.073 | 0.014 | 0.000 |  | CCI | 0.109 | 0.019 | 0.000 |  |
|  |  | Estimate | SE | p |  |  | Estimate | SE | p |  |
|  | (Intercept) | 3.189 | 0.058 | 0.000 |  | (Intercept) | 3.413 | 0.078 | 0.000 |  |
|  | ACE2 rs143695310 | -0.179 | 0.178 | 0.317 |  | ACE2 rs143695310 | -0.176 | 0.242 | 0.467 |  |
|  | Sex | -0.190 | 0.064 | 0.003 |  | Sex | -0.348 | 0.087 | 0.000 |  |
|  | CCI | 0.076 | 0.015 | 0.000 |  | CCI | 0.115 | 0.020 | 0.000 |  |
|  |  | Estimate | SE | p |  |  | Estimate | SE | p |  |
|  | (Intercept) | 3.232 | 0.068 | 0.000 |  | (Intercept) | 3.474 | 0.092 | 0.000 |  |
|  | ACE2 rs1996225 | -0.026 | 0.065 | 0.691 |  | ACE2 rs1996225 | -0.060 | 0.087 | 0.496 |  |
|  | Sex | -0.229 | 0.063 | 0.000 |  | Sex | -0.393 | 0.085 | 0.000 |  |
|  | CCI | 0.067 | 0.015 | 0.000 |  | CCI | 0.106 | 0.020 | 0.000 |  |
|  |  | Estimate | SE | p |  |  | Estimate | SE | p |  |
|  | (Intercept) | 3.181 | 0.068 | 0.000 |  | (Intercept) | 3.393 | 0.091 | 0.000 |  |
|  | ACE2 rs2074192 | 0.090 | 0.067 | 0.181 |  | ACE2 rs2074192 | 0.115 | 0.090 | 0.203 |  |
|  | Sex | -0.248 | 0.064 | 0.000 |  | Sex | -0.421 | 0.086 | 0.000 |  |
|  | CCI | 0.066 | 0.014 | 0.000 |  | CCI | 0.104 | 0.019 | 0.000 |  |
|  |  | Estimate | SE | p |  |  | Estimate | SE | p |  |
|  | (Intercept) | 3.098 | 0.096 | 0.000 |  | (Intercept) | 3.408 | 0.131 | 0.000 |  |
|  | ACE2 rs2106809 | 0.074 | 0.096 | 0.439 |  | ACE2 rs2106809 | -0.033 | 0.131 | 0.800 |  |
|  | Sex | -0.213 | 0.066 | 0.001 |  | Sex | -0.379 | 0.090 | 0.000 |  |
|  | CCI | 0.085 | 0.015 | 0.000 |  | CCI | 0.134 | 0.021 | 0.000 |  |
|  |  | Estimate | SE | p |  |  | Estimate | SE | p |  |
|  | (Intercept) | 3.233 | 0.084 | 0.000 |  | (Intercept) | 3.507 | 0.113 | 0.000 |  |
|  | ACE2 rs2285666 | -0.022 | 0.085 | 0.796 |  | ACE2 rs2285666 | -0.105 | 0.114 | 0.358 |  |
|  | Sex | -0.207 | 0.064 | 0.001 |  | Sex | -0.344 | 0.086 | 0.000 |  |
|  | CCI | 0.068 | 0.015 | 0.000 |  | CCI | 0.107 | 0.019 | 0.000 |  |
|  |  | Estimate | SE | p |  |  | Estimate | SE | p |  |
|  | (Intercept) | 3.119 | 0.153 | 0.000 |  | (Intercept) | 3.198 | 0.206 | 0.000 |  |
|  | ACE2 rs35803318 | 0.097 | 0.154 | 0.529 |  | ACE2 rs35803318 | 0.267 | 0.208 | 0.199 |  |
|  | Sex | -0.219 | 0.063 | 0.000 |  | Sex | -0.393 | 0.085 | 0.000 |  |
|  | CCI | 0.073 | 0.014 | 0.000 |  | CCI | 0.110 | 0.019 | 0.000 |  |
|  |  | Estimate | SE | p |  |  | Estimate | SE | p |  |
|  | (Intercept) | 3.292 | 0.328 | 0.000 |  | (Intercept) | 3.935 | 0.444 | 0.000 |  |
|  | ACE2 rs41303171 | -0.095 | 0.330 | 0.774 |  | ACE2 rs41303171 | -0.509 | 0.446 | 0.254 |  |
|  | Sex | -0.207 | 0.062 | 0.001 |  | Sex | -0.370 | 0.084 | 0.000 |  |
|  | CCI | 0.073 | 0.014 | 0.000 |  | CCI | 0.112 | 0.019 | 0.000 |  |
|  |  | Estimate | SE | p |  |  | Estimate | SE | p |  |
|  | (Intercept) | 3.247 | 0.076 | 0.000 |  | (Intercept) | 3.501 | 0.104 | 0.000 |  |
|  | ACE2 rs4646156 | -0.044 | 0.073 | 0.549 |  | ACE2 rs4646156 | -0.072 | 0.099 | 0.471 |  |
|  | Sex | -0.215 | 0.063 | 0.001 |  | Sex | -0.377 | 0.085 | 0.000 |  |
|  | CCI | 0.070 | 0.014 | 0.000 |  | CCI | 0.107 | 0.019 | 0.000 |  |
|  |  | Estimate | SE | p |  |  | Estimate | SE | p |  |
|  | (Intercept) | 3.270 | 0.129 | 0.000 |  | (Intercept) | 3.483 | 0.172 | 0.000 |  |
|  | ACE2 rs4646188 | -0.013 | 0.129 | 0.920 |  | ACE2 rs4646188 | -0.031 | 0.171 | 0.858 |  |
|  | Sex | -0.188 | 0.064 | 0.004 |  | Sex | -0.332 | 0.085 | 0.000 |  |
|  | CCI | 0.062 | 0.015 | 0.000 |  | CCI | 0.100 | 0.019 | 0.000 |  |
|  |  | Estimate | SE | p |  |  | Estimate | SE | p |  |
|  | (Intercept) | 3.246 | 0.076 | 0.000 |  | (Intercept) | 3.449 | 0.100 | 0.000 |  |
|  | ACE2 rs4830542 | -0.003 | 0.072 | 0.972 |  | ACE2 rs4830542 | -0.015 | 0.096 | 0.876 |  |
|  | Sex | -0.216 | 0.065 | 0.001 |  | Sex | -0.356 | 0.086 | 0.000 |  |
|  | CCI | 0.065 | 0.015 | 0.000 |  | CCI | 0.104 | 0.020 | 0.000 |  |
|  |  | Estimate | SE | p |  |  | Estimate | SE | p |  |
|  | (Intercept) | 2.860 | 0.167 | 0.000 |  | (Intercept) | 3.120 | 0.228 | 0.000 |  |
|  | ACE rs1799752 | 0.350 | 0.167 | 0.036 |  | ACE rs1799752 | 0.327 | 0.227 | 0.150 |  |
|  | Sex | -0.206 | 0.062 | 0.001 |  | Sex | -0.372 | 0.085 | 0.000 |  |
|  | CCI | 0.077 | 0.015 | 0.000 |  | CCI | 0.116 | 0.020 | 0.000 |  |
|  |  | Estimate | SE | p |  |  | Estimate | SE | p |  |
|  | (Intercept) | 3.184 | 0.096 | 0.000 |  | (Intercept) | 3.383 | 0.129 | 0.000 |  |
|  | ACE rs4291 | 0.025 | 0.090 | 0.783 |  | ACE rs4291 | 0.069 | 0.122 | 0.573 |  |
|  | Sex | -0.205 | 0.062 | 0.001 |  | Sex | -0.369 | 0.084 | 0.000 |  |
|  | CCI | 0.072 | 0.014 | 0.000 |  | CCI | 0.109 | 0.019 | 0.000 |  |
|  |  | Estimate | SE | p |  |  | Estimate | SE | p |  |
|  | (Intercept) | 3.271 | 0.080 | 0.000 |  | (Intercept) | 3.506 | 0.108 | 0.000 |  |
|  | ACE rs4343 | -0.076 | 0.073 | 0.297 |  | ACE rs4343 | -0.081 | 0.099 | 0.414 |  |
|  | Sex | -0.220 | 0.062 | 0.000 |  | Sex | -0.381 | 0.084 | 0.000 |  |
|  | CCI | 0.073 | 0.015 | 0.000 |  | CCI | 0.112 | 0.020 | 0.000 |  |
|  |  | Estimate | SE | p |  |  | Estimate | SE | p |  |
|  | (Intercept) | 3.275 | 0.092 | 0.000 |  | (Intercept) | 3.448 | 0.125 | 0.000 |  |
|  | ADAM17 rs12692386 | -0.086 | 0.086 | 0.321 |  | ADAM17 rs12692386 | -0.008 | 0.117 | 0.945 |  |
|  | Sex | -0.210 | 0.062 | 0.001 |  | Sex | -0.375 | 0.084 | 0.000 |  |
|  | CCI | 0.076 | 0.014 | 0.000 |  | CCI | 0.113 | 0.020 | 0.000 |  |
|  |  | Estimate | SE | p |  |  | Estimate | SE | p |  |
|  | (Intercept) | 3.390 | 0.142 | 0.000 |  | (Intercept) | 3.543 | 0.194 | 0.000 |  |
|  | ADAM17 rs55790676 | -0.177 | 0.137 | 0.198 |  | ADAM17 rs55790676 | -0.094 | 0.187 | 0.614 |  |
|  | Sex | -0.218 | 0.061 | 0.000 |  | Sex | -0.376 | 0.084 | 0.000 |  |
|  | CCI | 0.067 | 0.014 | 0.000 |  | CCI | 0.104 | 0.019 | 0.000 |  |
|  |  | Estimate | SE | p |  |  | Estimate | SE | p |  |
|  | (Intercept) | 3.245 | 0.076 | 0.000 |  | (Intercept) | 3.480 | 0.102 | 0.000 |  |
|  | AGT rs699 | -0.041 | 0.071 | 0.568 |  | AGT rs699 | -0.040 | 0.096 | 0.680 |  |
|  | Sex | -0.219 | 0.062 | 0.000 |  | Sex | -0.388 | 0.084 | 0.000 |  |
|  | CCI | 0.070 | 0.014 | 0.000 |  | CCI | 0.107 | 0.020 | 0.000 |  |
|  |  | Estimate | SE | p |  |  | Estimate | SE | p |  |
|  | (Intercept) | 2.973 | 0.253 | 0.000 |  | (Intercept) | 2.991 | 0.343 | 0.000 |  |
|  | APOE rs429358 | 0.234 | 0.252 | 0.352 |  | APOE rs429358 | 0.460 | 0.341 | 0.178 |  |
|  | Sex | -0.200 | 0.062 | 0.001 |  | Sex | -0.364 | 0.084 | 0.000 |  |
|  | CCI | 0.073 | 0.014 | 0.000 |  | CCI | 0.109 | 0.020 | 0.000 |  |
|  |  | Estimate | SE | p |  |  | Estimate | SE | p |  |
|  | (Intercept) | 3.666 | 0.389 | 0.000 |  | (Intercept) | 3.621 | 0.529 | 0.000 |  |
|  | APOE rs7412 | -0.464 | 0.387 | 0.231 |  | APOE rs7412 | -0.180 | 0.526 | 0.731 |  |
|  | Sex | -0.209 | 0.062 | 0.001 |  | Sex | -0.371 | 0.084 | 0.000 |  |
|  | CCI | 0.073 | 0.014 | 0.000 |  | CCI | 0.109 | 0.019 | 0.000 |  |
|  |  | Estimate | SE | p |  |  | Estimate | SE | p |  |
|  | (Intercept) | 3.233 | 0.102 | 0.000 |  | (Intercept) | 3.559 | 0.137 | 0.000 |  |
|  | BSG rs8259 | -0.034 | 0.099 | 0.729 |  | BSG rs8259 | -0.146 | 0.134 | 0.277 |  |
|  | Sex | -0.206 | 0.062 | 0.001 |  | Sex | -0.368 | 0.084 | 0.000 |  |
|  | CCI | 0.073 | 0.014 | 0.000 |  | CCI | 0.113 | 0.019 | 0.000 |  |
|  |  | Estimate | SE | p |  |  | Estimate | SE | p |  |
|  | (Intercept) | 3.385 | 0.103 | 0.000 |  | (Intercept) | 3.606 | 0.141 | 0.000 |  |
|  | CCL2 rs1024611 | -0.192 | 0.096 | 0.045 |  | CCL2 rs1024611 | -0.176 | 0.131 | 0.179 |  |
|  | Sex | -0.219 | 0.062 | 0.000 |  | Sex | -0.377 | 0.085 | 0.000 |  |
|  | CCI | 0.068 | 0.014 | 0.000 |  | CCI | 0.105 | 0.020 | 0.000 |  |
|  |  | Estimate | SE | p |  |  | Estimate | SE | p |  |
|  | (Intercept) | 3.425 | 0.150 | 0.000 |  | (Intercept) | 3.748 | 0.203 | 0.000 |  |
|  | CCL5 rs2107538 | -0.206 | 0.146 | 0.157 |  | CCL5 rs2107538 | -0.297 | 0.197 | 0.133 |  |
|  | Sex | -0.235 | 0.062 | 0.000 |  | Sex | -0.404 | 0.083 | 0.000 |  |
|  | CCI | 0.068 | 0.014 | 0.000 |  | CCI | 0.103 | 0.019 | 0.000 |  |
|  |  | Estimate | SE | p |  |  | Estimate | SE | p |  |
|  | (Intercept) | 2.854 | 0.118 | 0.000 |  | (Intercept) | 2.987 | 0.163 | 0.000 |  |
|  | CD14 rs2569190 | 0.016 | 0.103 | 0.879 |  | CD14 rs2569190 | 0.055 | 0.143 | 0.699 |  |
|  | Sex | -0.231 | 0.085 | 0.006 |  | Sex | -0.397 | 0.117 | 0.001 |  |
|  | CCI | 0.135 | 0.020 | 0.000 |  | CCI | 0.211 | 0.028 | 0.000 |  |
|  |  | Estimate | SE | p |  |  | Estimate | SE | p |  |
|  | (Intercept) | 3.271 | 0.094 | 0.000 |  | (Intercept) | 3.430 | 0.124 | 0.000 |  |
|  | CD69 rs11052877 | -0.057 | 0.088 | 0.515 |  | CD69 rs11052877 | -0.016 | 0.116 | 0.889 |  |
|  | Sex | -0.202 | 0.063 | 0.001 |  | Sex | -0.341 | 0.083 | 0.000 |  |
|  | CCI | 0.064 | 0.015 | 0.000 |  | CCI | 0.101 | 0.019 | 0.000 |  |
|  |  | Estimate | SE | p |  |  | Estimate | SE | p |  |
|  | (Intercept) | 3.155 | 0.092 | 0.000 |  | (Intercept) | 3.357 | 0.126 | 0.000 |  |
|  | CLEC2D rs1560011 | 0.071 | 0.085 | 0.401 |  | CLEC2D rs1560011 | 0.112 | 0.115 | 0.332 |  |
|  | Sex | -0.214 | 0.062 | 0.001 |  | Sex | -0.380 | 0.084 | 0.000 |  |
|  | CCI | 0.069 | 0.014 | 0.000 |  | CCI | 0.108 | 0.020 | 0.000 |  |
|  |  | Estimate | SE | p |  |  | Estimate | SE | p |  |
|  | (Intercept) | 3.112 | 0.101 | 0.000 |  | (Intercept) | 3.353 | 0.137 | 0.000 |  |
|  | CRP rs1130864 | 0.111 | 0.098 | 0.258 |  | CRP rs1130864 | 0.105 | 0.132 | 0.427 |  |
|  | Sex | -0.212 | 0.062 | 0.001 |  | Sex | -0.376 | 0.084 | 0.000 |  |
|  | CCI | 0.072 | 0.014 | 0.000 |  | CCI | 0.110 | 0.020 | 0.000 |  |
|  |  | Estimate | SE | p |  |  | Estimate | SE | p |  |
|  | (Intercept) | 3.248 | 0.096 | 0.000 |  | (Intercept) | 3.468 | 0.131 | 0.000 |  |
|  | CSF3 rs2227322 | -0.037 | 0.091 | 0.685 |  | CSF3 rs2227322 | -0.023 | 0.123 | 0.850 |  |
|  | Sex | -0.220 | 0.062 | 0.000 |  | Sex | -0.377 | 0.084 | 0.000 |  |
|  | CCI | 0.069 | 0.014 | 0.000 |  | CCI | 0.106 | 0.019 | 0.000 |  |
|  |  | Estimate | SE | p |  |  | Estimate | SE | p |  |
|  | (Intercept) | 3.400 | 0.113 | 0.000 |  | (Intercept) | 3.531 | 0.152 | 0.000 |  |
|  | CXCL1 rs2071425 | -0.212 | 0.107 | 0.049 |  | CXCL1 rs2071425 | -0.116 | 0.145 | 0.423 |  |
|  | Sex | -0.220 | 0.062 | 0.000 |  | Sex | -0.369 | 0.084 | 0.000 |  |
|  | CCI | 0.069 | 0.015 | 0.000 |  | CCI | 0.108 | 0.020 | 0.000 |  |
|  |  | Estimate | SE | p |  |  | Estimate | SE | p |  |
|  | (Intercept) | 3.108 | 0.166 | 0.000 |  | (Intercept) | 3.156 | 0.219 | 0.000 |  |
|  | CYP2C19 rs12248560 | 0.133 | 0.160 | 0.407 |  | CYP2C19 rs12248560 | 0.277 | 0.212 | 0.191 |  |
|  | Sex | -0.184 | 0.064 | 0.004 |  | Sex | -0.316 | 0.084 | 0.000 |  |
|  | CCI | 0.064 | 0.015 | 0.000 |  | CCI | 0.103 | 0.019 | 0.000 |  |
|  |  | Estimate | SE | p |  |  | Estimate | SE | p |  |
|  | (Intercept) | 3.329 | 0.144 | 0.000 |  | (Intercept) | 3.823 | 0.195 | 0.000 |  |
|  | CYP2C19 rs4244285 | -0.112 | 0.139 | 0.421 |  | CYP2C19 rs4244285 | -0.394 | 0.188 | 0.037 |  |
|  | Sex | -0.195 | 0.063 | 0.002 |  | Sex | -0.342 | 0.085 | 0.000 |  |
|  | CCI | 0.065 | 0.015 | 0.000 |  | CCI | 0.103 | 0.020 | 0.000 |  |
|  |  | Estimate | SE | p |  |  | Estimate | SE | p |  |
|  | (Intercept) | 3.472 | 0.605 | 0.000 |  | (Intercept) | 4.466 | 0.799 | 0.000 |  |
|  | CYP2C9 rs1057910 | -0.247 | 0.604 | 0.682 |  | CYP2C9 rs1057910 | -1.048 | 0.798 | 0.189 |  |
|  | Sex | -0.203 | 0.063 | 0.001 |  | Sex | -0.344 | 0.083 | 0.000 |  |
|  | CCI | 0.065 | 0.015 | 0.000 |  | CCI | 0.103 | 0.019 | 0.000 |  |
|  |  | Estimate | SE | p |  |  | Estimate | SE | p |  |
|  | (Intercept) | 3.345 | 0.213 | 0.000 |  | (Intercept) | 3.657 | 0.290 | 0.000 |  |
|  | CYP2C9 rs1799853 | -0.125 | 0.210 | 0.551 |  | CYP2C9 rs1799853 | -0.204 | 0.285 | 0.474 |  |
|  | Sex | -0.229 | 0.061 | 0.000 |  | Sex | -0.396 | 0.084 | 0.000 |  |
|  | CCI | 0.068 | 0.014 | 0.000 |  | CCI | 0.105 | 0.019 | 0.000 |  |
|  |  | Estimate | SE | p |  |  | Estimate | SE | p |  |
|  | (Intercept) | 3.317 | 0.609 | 0.000 |  | (Intercept) | 3.234 | 0.828 | 0.000 |  |
|  | CYP3A4 rs35599367 | -0.086 | 0.606 | 0.887 |  | CYP3A4 rs35599367 | 0.233 | 0.823 | 0.777 |  |
|  | Sex | -0.230 | 0.062 | 0.000 |  | Sex | -0.391 | 0.084 | 0.000 |  |
|  | CCI | 0.066 | 0.014 | 0.000 |  | CCI | 0.103 | 0.019 | 0.000 |  |
|  |  | Estimate | SE | p |  |  | Estimate | SE | p |  |
|  | (Intercept) | 3.233 | 0.057 | 0.000 |  | (Intercept) | 3.430 | 0.076 | 0.000 |  |
|  | CYP3A4 rs67666821 | -0.456 | 0.382 | 0.233 |  | CYP3A4 rs67666821 | -0.791 | 0.510 | 0.122 |  |
|  | Sex | -0.226 | 0.063 | 0.000 |  | Sex | -0.364 | 0.084 | 0.000 |  |
|  | CCI | 0.068 | 0.015 | 0.000 |  | CCI | 0.110 | 0.020 | 0.000 |  |
|  |  | Estimate | SE | p |  |  | Estimate | SE | p |  |
|  | (Intercept) | 2.774 | 0.226 | 0.000 |  | (Intercept) | 2.921 | 0.306 | 0.000 |  |
|  | CYP3A5 rs776746 | 0.457 | 0.222 | 0.040 |  | CYP3A5 rs776746 | 0.545 | 0.301 | 0.071 |  |
|  | Sex | -0.217 | 0.062 | 0.000 |  | Sex | -0.378 | 0.083 | 0.000 |  |
|  | CCI | 0.067 | 0.014 | 0.000 |  | CCI | 0.103 | 0.019 | 0.000 |  |
|  |  | Estimate | SE | p |  |  | Estimate | SE | p |  |
|  | (Intercept) | 3.261 | 0.079 | 0.000 |  | (Intercept) | 3.483 | 0.107 | 0.000 |  |
|  | CYP4V2 rs13146272 | -0.056 | 0.073 | 0.448 |  | CYP4V2 rs13146272 | -0.037 | 0.100 | 0.714 |  |
|  | Sex | -0.213 | 0.061 | 0.001 |  | Sex | -0.381 | 0.083 | 0.000 |  |
|  | CCI | 0.069 | 0.014 | 0.000 |  | CCI | 0.106 | 0.019 | 0.000 |  |
|  |  | Estimate | SE | p |  |  | Estimate | SE | p |  |
|  | (Intercept) | 3.045 | 0.603 | 0.000 |  | (Intercept) | 3.088 | 0.798 | 0.000 |  |
|  | DPP4 rs116302758 | 0.181 | 0.603 | 0.764 |  | DPP4 rs116302758 | 0.346 | 0.798 | 0.665 |  |
|  | Sex | -0.225 | 0.063 | 0.000 |  | Sex | -0.385 | 0.083 | 0.000 |  |
|  | CCI | 0.068 | 0.015 | 0.000 |  | CCI | 0.105 | 0.019 | 0.000 |  |
|  |  | Estimate | SE | p |  |  | Estimate | SE | p |  |
|  | (Intercept) | 3.104 | 0.102 | 0.000 |  | (Intercept) | 3.182 | 0.138 | 0.000 |  |
|  | DPP4 rs17574 | 0.121 | 0.098 | 0.218 |  | DPP4 rs17574 | 0.306 | 0.133 | 0.021 |  |
|  | Sex | -0.217 | 0.062 | 0.000 |  | Sex | -0.393 | 0.084 | 0.000 |  |
|  | CCI | 0.073 | 0.014 | 0.000 |  | CCI | 0.111 | 0.019 | 0.000 |  |
|  |  | Estimate | SE | p |  |  | Estimate | SE | p |  |
|  | (Intercept) | 3.261 | 0.059 | 0.000 |  | (Intercept) | 3.454 | 0.078 | 0.000 |  |
|  | DPP4 rs56179129 | -0.106 | 0.235 | 0.652 |  | DPP4 rs56179129 | 0.020 | 0.314 | 0.950 |  |
|  | Sex | -0.181 | 0.064 | 0.005 |  | Sex | -0.323 | 0.085 | 0.000 |  |
|  | CCI | 0.060 | 0.015 | 0.000 |  | CCI | 0.100 | 0.020 | 0.000 |  |
|  |  | Estimate | SE | p |  |  | Estimate | SE | p |  |
|  | (Intercept) | 3.214 | 0.077 | 0.000 |  | (Intercept) | 3.493 | 0.103 | 0.000 |  |
|  | ENOX1 rs9594987 | 0.018 | 0.071 | 0.801 |  | ENOX1 rs9594987 | -0.058 | 0.095 | 0.543 |  |
|  | Sex | -0.230 | 0.063 | 0.000 |  | Sex | -0.386 | 0.084 | 0.000 |  |
|  | CCI | 0.067 | 0.015 | 0.000 |  | CCI | 0.102 | 0.020 | 0.000 |  |
|  |  | Estimate | SE | p |  |  | Estimate | SE | p |  |
|  | (Intercept) | 3.336 | 0.100 | 0.000 |  | (Intercept) | 3.591 | 0.135 | 0.000 |  |
|  | EPHX1 rs1051740 | -0.127 | 0.096 | 0.188 |  | EPHX1 rs1051740 | -0.173 | 0.131 | 0.187 |  |
|  | Sex | -0.214 | 0.061 | 0.001 |  | Sex | -0.358 | 0.083 | 0.000 |  |
|  | CCI | 0.065 | 0.014 | 0.000 |  | CCI | 0.104 | 0.019 | 0.000 |  |
|  |  | Estimate | SE | p |  |  | Estimate | SE | p |  |
|  | (Intercept) | 3.192 | 0.080 | 0.000 |  | (Intercept) | 3.336 | 0.109 | 0.000 |  |
|  | F11 rs2036914 | 0.029 | 0.072 | 0.685 |  | F11 rs2036914 | 0.147 | 0.098 | 0.132 |  |
|  | Sex | -0.227 | 0.062 | 0.000 |  | Sex | -0.402 | 0.083 | 0.000 |  |
|  | CCI | 0.070 | 0.014 | 0.000 |  | CCI | 0.109 | 0.019 | 0.000 |  |
|  |  | Estimate | SE | p |  |  | Estimate | SE | p |  |
|  | (Intercept) | 3.241 | 0.091 | 0.000 |  | (Intercept) | 3.534 | 0.123 | 0.000 |  |
|  | F11 rs2289252 | -0.047 | 0.086 | 0.583 |  | F11 rs2289252 | -0.114 | 0.116 | 0.328 |  |
|  | Sex | -0.205 | 0.062 | 0.001 |  | Sex | -0.368 | 0.084 | 0.000 |  |
|  | CCI | 0.075 | 0.014 | 0.000 |  | CCI | 0.112 | 0.019 | 0.000 |  |
|  |  | Estimate | SE | p |  |  | Estimate | SE | p |  |
|  | (Intercept) | 3.210 | 0.158 | 0.000 |  | (Intercept) | 3.499 | 0.209 | 0.000 |  |
|  | FGG rs2066865 | 0.015 | 0.154 | 0.922 |  | FGG rs2066865 | -0.081 | 0.204 | 0.691 |  |
|  | Sex | -0.202 | 0.063 | 0.001 |  | Sex | -0.349 | 0.084 | 0.000 |  |
|  | CCI | 0.065 | 0.015 | 0.000 |  | CCI | 0.105 | 0.019 | 0.000 |  |
|  |  | Estimate | SE | p |  |  | Estimate | SE | p |  |
|  | (Intercept) | 3.213 | 0.056 | 0.000 |  | (Intercept) | 3.449 | 0.076 | 0.000 |  |
|  | G6PD rs1050828 | 0.117 | 0.433 | 0.787 |  | G6PD rs1050828 | -0.026 | 0.586 | 0.965 |  |
|  | Sex | -0.219 | 0.062 | 0.000 |  | Sex | -0.386 | 0.084 | 0.000 |  |
|  | CCI | 0.069 | 0.014 | 0.000 |  | CCI | 0.106 | 0.019 | 0.000 |  |
|  |  | Estimate | SE | p |  |  | Estimate | SE | p |  |
|  | (Intercept) | 3.208 | 0.056 | 0.000 |  | (Intercept) | 3.447 | 0.076 | 0.000 |  |
|  | G6PD rs1050829 | 0.249 | 0.388 | 0.522 |  | G6PD rs1050829 | 0.099 | 0.526 | 0.850 |  |
|  | Sex | -0.217 | 0.062 | 0.000 |  | Sex | -0.389 | 0.084 | 0.000 |  |
|  | CCI | 0.073 | 0.014 | 0.000 |  | CCI | 0.110 | 0.019 | 0.000 |  |
|  |  | Estimate | SE | p |  |  | Estimate | SE | p |  |
|  | (Intercept) | 3.324 | 0.121 | 0.000 |  | (Intercept) | 3.482 | 0.166 | 0.000 |  |
|  | GC rs4588 | -0.102 | 0.116 | 0.380 |  | GC rs4588 | -0.012 | 0.159 | 0.940 |  |
|  | Sex | -0.223 | 0.061 | 0.000 |  | Sex | -0.391 | 0.084 | 0.000 |  |
|  | CCI | 0.069 | 0.014 | 0.000 |  | CCI | 0.107 | 0.020 | 0.000 |  |
|  |  | Estimate | SE | p |  |  | Estimate | SE | p |  |
|  | (Intercept) | 3.266 | 0.082 | 0.000 |  | (Intercept) | 3.463 | 0.111 | 0.000 |  |
|  | GC rs7041 | -0.077 | 0.076 | 0.309 |  | GC rs7041 | -0.027 | 0.102 | 0.790 |  |
|  | Sex | -0.207 | 0.062 | 0.001 |  | Sex | -0.375 | 0.083 | 0.000 |  |
|  | CCI | 0.073 | 0.014 | 0.000 |  | CCI | 0.109 | 0.019 | 0.000 |  |
|  |  | Estimate | SE | p |  |  | Estimate | SE | p |  |
|  | (Intercept) | 3.247 | 0.057 | 0.000 |  | (Intercept) | 3.493 | 0.077 | 0.000 |  |
|  | HCP5 rs2395029 | -0.363 | 0.154 | 0.019 |  | HCP5 rs2395029 | -0.595 | 0.209 | 0.005 |  |
|  | Sex | -0.238 | 0.061 | 0.000 |  | Sex | -0.400 | 0.083 | 0.000 |  |
|  | CCI | 0.067 | 0.014 | 0.000 |  | CCI | 0.103 | 0.019 | 0.000 |  |
|  |  | Estimate | SE | p |  |  | Estimate | SE | p |  |
|  | (Intercept) | 3.051 | 0.091 | 0.000 |  | (Intercept) | 3.322 | 0.124 | 0.000 |  |
|  | HMOX1 rs2071746 | 0.177 | 0.083 | 0.034 |  | HMOX1 rs2071746 | 0.140 | 0.113 | 0.216 |  |
|  | Sex | -0.201 | 0.062 | 0.001 |  | Sex | -0.369 | 0.084 | 0.000 |  |
|  | CCI | 0.075 | 0.014 | 0.000 |  | CCI | 0.113 | 0.019 | 0.000 |  |
|  |  | Estimate | SE | p |  |  | Estimate | SE | p |  |
|  | (Intercept) | 2.739 | 0.362 | 0.000 |  | (Intercept) | 2.708 | 0.494 | 0.000 |  |
|  | IFITM3 rs12252 | 0.623 | 0.359 | 0.083 |  | IFITM3 rs12252 | 0.851 | 0.490 | 0.083 |  |
|  | Sex | -0.084 | 0.066 | 0.205 |  | Sex | -0.216 | 0.090 | 0.017 |  |
|  | CCI | 0.043 | 0.015 | 0.004 |  | CCI | 0.085 | 0.020 | 0.000 |  |
|  |  | Estimate | SE | p |  |  | Estimate | SE | p |  |
|  | (Intercept) | 3.034 | 0.103 | 0.000 |  | (Intercept) | 3.188 | 0.139 | 0.000 |  |
|  | IFNL4 rs12979860 | 0.193 | 0.096 | 0.046 |  | IFNL4 rs12979860 | 0.287 | 0.130 | 0.028 |  |
|  | Sex | -0.216 | 0.062 | 0.001 |  | Sex | -0.392 | 0.083 | 0.000 |  |
|  | CCI | 0.073 | 0.014 | 0.000 |  | CCI | 0.110 | 0.019 | 0.000 |  |
|  |  | Estimate | SE | p |  |  | Estimate | SE | p |  |
|  | (Intercept) | 3.237 | 0.107 | 0.000 |  | (Intercept) | 3.428 | 0.145 | 0.000 |  |
|  | IL10 rs1800871 | -0.009 | 0.103 | 0.928 |  | IL10 rs1800871 | 0.040 | 0.140 | 0.775 |  |
|  | Sex | -0.227 | 0.062 | 0.000 |  | Sex | -0.394 | 0.084 | 0.000 |  |
|  | CCI | 0.067 | 0.014 | 0.000 |  | CCI | 0.104 | 0.020 | 0.000 |  |
|  |  | Estimate | SE | p |  |  | Estimate | SE | p |  |
|  | (Intercept) | 3.138 | 0.094 | 0.000 |  | (Intercept) | 3.298 | 0.128 | 0.000 |  |
|  | IL10 rs1800896 | 0.072 | 0.087 | 0.406 |  | IL10 rs1800896 | 0.162 | 0.117 | 0.167 |  |
|  | Sex | -0.215 | 0.062 | 0.001 |  | Sex | -0.387 | 0.083 | 0.000 |  |
|  | CCI | 0.075 | 0.014 | 0.000 |  | CCI | 0.114 | 0.019 | 0.000 |  |
|  |  | Estimate | SE | p |  |  | Estimate | SE | p |  |
|  | (Intercept) | 3.075 | 0.175 | 0.000 |  | (Intercept) | 3.379 | 0.239 | 0.000 |  |
|  | IL13 rs1800925 | 0.144 | 0.171 | 0.401 |  | IL13 rs1800925 | 0.075 | 0.233 | 0.746 |  |
|  | Sex | -0.220 | 0.061 | 0.000 |  | Sex | -0.389 | 0.084 | 0.000 |  |
|  | CCI | 0.071 | 0.014 | 0.000 |  | CCI | 0.108 | 0.020 | 0.000 |  |
|  |  | Estimate | SE | p |  |  | Estimate | SE | p |  |
|  | (Intercept) | 3.190 | 0.113 | 0.000 |  | (Intercept) | 3.374 | 0.152 | 0.000 |  |
|  | IL17A rs2275913 | 0.041 | 0.108 | 0.706 |  | IL17A rs2275913 | 0.089 | 0.145 | 0.541 |  |
|  | Sex | -0.233 | 0.062 | 0.000 |  | Sex | -0.401 | 0.084 | 0.000 |  |
|  | CCI | 0.067 | 0.015 | 0.000 |  | CCI | 0.105 | 0.020 | 0.000 |  |
|  |  | Estimate | SE | p |  |  | Estimate | SE | p |  |
|  | (Intercept) | 3.286 | 0.156 | 0.000 |  | (Intercept) | 3.589 | 0.211 | 0.000 |  |
|  | IL17A rs3819025 | -0.074 | 0.151 | 0.623 |  | IL17A rs3819025 | -0.142 | 0.205 | 0.489 |  |
|  | Sex | -0.219 | 0.062 | 0.000 |  | Sex | -0.391 | 0.084 | 0.000 |  |
|  | CCI | 0.070 | 0.014 | 0.000 |  | CCI | 0.108 | 0.019 | 0.000 |  |
|  |  | Estimate | SE | p |  |  | Estimate | SE | p |  |
|  | (Intercept) | 3.189 | 0.091 | 0.000 |  | (Intercept) | 3.443 | 0.123 | 0.000 |  |
|  | IL1B rs1143627 | 0.018 | 0.084 | 0.831 |  | IL1B rs1143627 | -0.003 | 0.114 | 0.976 |  |
|  | Sex | -0.206 | 0.062 | 0.001 |  | Sex | -0.376 | 0.084 | 0.000 |  |
|  | CCI | 0.073 | 0.014 | 0.000 |  | CCI | 0.110 | 0.019 | 0.000 |  |
|  |  | Estimate | SE | p |  |  | Estimate | SE | p |  |
|  | (Intercept) | 3.345 | 0.155 | 0.000 |  | (Intercept) | 3.568 | 0.211 | 0.000 |  |
|  | IL1B rs1143634 | -0.136 | 0.150 | 0.365 |  | IL1B rs1143634 | -0.126 | 0.204 | 0.537 |  |
|  | Sex | -0.222 | 0.061 | 0.000 |  | Sex | -0.391 | 0.083 | 0.000 |  |
|  | CCI | 0.070 | 0.014 | 0.000 |  | CCI | 0.109 | 0.019 | 0.000 |  |
|  |  | Estimate | SE | p |  |  | Estimate | SE | p |  |
|  | (Intercept) | 3.189 | 0.148 | 0.000 |  | (Intercept) | 3.533 | 0.201 | 0.000 |  |
|  | IL1RN rs315952 | 0.009 | 0.144 | 0.950 |  | IL1RN rs315952 | -0.104 | 0.195 | 0.595 |  |
|  | Sex | -0.200 | 0.062 | 0.001 |  | Sex | -0.364 | 0.084 | 0.000 |  |
|  | CCI | 0.074 | 0.014 | 0.000 |  | CCI | 0.112 | 0.020 | 0.000 |  |
|  |  | Estimate | SE | p |  |  | Estimate | SE | p |  |
|  | (Intercept) | 3.049 | 0.170 | 0.000 |  | (Intercept) | 3.166 | 0.230 | 0.000 |  |
|  | IL6R rs11265618 | 0.164 | 0.167 | 0.327 |  | IL6R rs11265618 | 0.289 | 0.226 | 0.201 |  |
|  | Sex | -0.211 | 0.062 | 0.001 |  | Sex | -0.377 | 0.084 | 0.000 |  |
|  | CCI | 0.073 | 0.014 | 0.000 |  | CCI | 0.110 | 0.020 | 0.000 |  |
|  |  | Estimate | SE | p |  |  | Estimate | SE | p |  |
|  | (Intercept) | 3.241 | 0.146 | 0.000 |  | (Intercept) | 3.489 | 0.198 | 0.000 |  |
|  | IL6R rs12083537 | -0.032 | 0.144 | 0.824 |  | IL6R rs12083537 | -0.043 | 0.195 | 0.825 |  |
|  | Sex | -0.209 | 0.062 | 0.001 |  | Sex | -0.371 | 0.084 | 0.000 |  |
|  | CCI | 0.072 | 0.014 | 0.000 |  | CCI | 0.108 | 0.019 | 0.000 |  |
|  |  | Estimate | SE | p |  |  | Estimate | SE | p |  |
|  | (Intercept) | 3.255 | 0.094 | 0.000 |  | (Intercept) | 3.466 | 0.127 | 0.000 |  |
|  | IL6R rs2228145 | -0.039 | 0.086 | 0.653 |  | IL6R rs2228145 | -0.025 | 0.117 | 0.833 |  |
|  | Sex | -0.216 | 0.062 | 0.001 |  | Sex | -0.366 | 0.084 | 0.000 |  |
|  | CCI | 0.067 | 0.014 | 0.000 |  | CCI | 0.104 | 0.019 | 0.000 |  |
|  |  | Estimate | SE | p |  |  | Estimate | SE | p |  |
|  | (Intercept) | 3.001 | 0.180 | 0.000 |  | (Intercept) | 3.013 | 0.245 | 0.000 |  |
|  | IL6R rs4329505 | 0.224 | 0.177 | 0.208 |  | IL6R rs4329505 | 0.452 | 0.241 | 0.061 |  |
|  | Sex | -0.217 | 0.061 | 0.000 |  | Sex | -0.379 | 0.084 | 0.000 |  |
|  | CCI | 0.068 | 0.014 | 0.000 |  | CCI | 0.105 | 0.019 | 0.000 |  |
|  |  | Estimate | SE | p |  |  | Estimate | SE | p |  |
|  | (Intercept) | 3.282 | 0.089 | 0.000 |  | (Intercept) | 3.544 | 0.121 | 0.000 |  |
|  | IL6R rs7529229 | -0.070 | 0.081 | 0.393 |  | IL6R rs7529229 | -0.104 | 0.111 | 0.346 |  |
|  | Sex | -0.223 | 0.062 | 0.000 |  | Sex | -0.380 | 0.084 | 0.000 |  |
|  | CCI | 0.069 | 0.014 | 0.000 |  | CCI | 0.104 | 0.019 | 0.000 |  |
|  |  | Estimate | SE | p |  |  | Estimate | SE | p |  |
|  | (Intercept) | 3.060 | 0.615 | 0.000 |  | (Intercept) | 3.147 | 0.834 | 0.000 |  |
|  | IL6 rs1800795 | 0.146 | 0.613 | 0.812 |  | IL6 rs1800795 | 0.295 | 0.831 | 0.723 |  |
|  | Sex | -0.205 | 0.062 | 0.001 |  | Sex | -0.365 | 0.084 | 0.000 |  |
|  | CCI | 0.072 | 0.014 | 0.000 |  | CCI | 0.109 | 0.019 | 0.000 |  |
|  |  | Estimate | SE | p |  |  | Estimate | SE | p |  |
|  | (Intercept) | 3.055 | 0.108 | 0.000 |  | (Intercept) | 3.349 | 0.146 | 0.000 |  |
|  | IL6 rs1800796 | 0.167 | 0.102 | 0.103 |  | IL6 rs1800796 | 0.102 | 0.139 | 0.463 |  |
|  | Sex | -0.203 | 0.062 | 0.001 |  | Sex | -0.365 | 0.084 | 0.000 |  |
|  | CCI | 0.072 | 0.014 | 0.000 |  | CCI | 0.109 | 0.020 | 0.000 |  |
|  |  | Estimate | SE | p |  |  | Estimate | SE | p |  |
|  | (Intercept) | 3.424 | 0.155 | 0.000 |  | (Intercept) | 3.747 | 0.210 | 0.000 |  |
|  | IL6 rs1818879 | -0.227 | 0.151 | 0.133 |  | IL6 rs1818879 | -0.316 | 0.205 | 0.124 |  |
|  | Sex | -0.211 | 0.062 | 0.001 |  | Sex | -0.384 | 0.084 | 0.000 |  |
|  | CCI | 0.074 | 0.014 | 0.000 |  | CCI | 0.112 | 0.019 | 0.000 |  |
|  |  | Estimate | SE | p |  |  | Estimate | SE | p |  |
|  | (Intercept) | 3.209 | 0.086 | 0.000 |  | (Intercept) | 3.475 | 0.116 | 0.000 |  |
|  | KCNIP1 rs703505 | -0.005 | 0.079 | 0.952 |  | KCNIP1 rs703505 | -0.040 | 0.107 | 0.706 |  |
|  | Sex | -0.212 | 0.062 | 0.001 |  | Sex | -0.383 | 0.084 | 0.000 |  |
|  | CCI | 0.075 | 0.014 | 0.000 |  | CCI | 0.113 | 0.019 | 0.000 |  |
|  |  | Estimate | SE | p |  |  | Estimate | SE | p |  |
|  | (Intercept) | 3.757 | 0.498 | 0.000 |  | (Intercept) | 3.617 | 0.679 | 0.000 |  |
|  | LZTFL1 rs35044562 | -0.536 | 0.497 | 0.281 |  | LZTFL1 rs35044562 | -0.159 | 0.677 | 0.814 |  |
|  | Sex | -0.217 | 0.062 | 0.000 |  | Sex | -0.381 | 0.084 | 0.000 |  |
|  | CCI | 0.066 | 0.014 | 0.000 |  | CCI | 0.104 | 0.020 | 0.000 |  |
|  |  | Estimate | SE | p |  |  | Estimate | SE | p |  |
|  | (Intercept) | 3.371 | 0.118 | 0.000 |  | (Intercept) | 3.705 | 0.160 | 0.000 |  |
|  | MTHFR rs1801131 | -0.180 | 0.113 | 0.112 |  | MTHFR rs1801131 | -0.284 | 0.154 | 0.065 |  |
|  | Sex | -0.199 | 0.062 | 0.001 |  | Sex | -0.362 | 0.084 | 0.000 |  |
|  | CCI | 0.072 | 0.014 | 0.000 |  | CCI | 0.109 | 0.019 | 0.000 |  |
|  |  | Estimate | SE | p |  |  | Estimate | SE | p |  |
|  | (Intercept) | 3.315 | 0.084 | 0.000 |  | (Intercept) | 3.519 | 0.114 | 0.000 |  |
|  | MTHFR rs1801133 | -0.147 | 0.078 | 0.062 |  | MTHFR rs1801133 | -0.107 | 0.106 | 0.313 |  |
|  | Sex | -0.198 | 0.062 | 0.001 |  | Sex | -0.368 | 0.084 | 0.000 |  |
|  | CCI | 0.075 | 0.014 | 0.000 |  | CCI | 0.113 | 0.020 | 0.000 |  |
|  |  | Estimate | SE | p |  |  | Estimate | SE | p |  |
|  | (Intercept) | 3.330 | 0.080 | 0.000 |  | (Intercept) | 3.548 | 0.109 | 0.000 |  |
|  | MX1 rs469390 | -0.145 | 0.073 | 0.046 |  | MX1 rs469390 | -0.126 | 0.099 | 0.204 |  |
|  | Sex | -0.217 | 0.061 | 0.000 |  | Sex | -0.374 | 0.083 | 0.000 |  |
|  | CCI | 0.068 | 0.014 | 0.000 |  | CCI | 0.105 | 0.019 | 0.000 |  |
|  |  | Estimate | SE | p |  |  | Estimate | SE | p |  |
|  | (Intercept) | 3.364 | 0.088 | 0.000 |  | (Intercept) | 3.691 | 0.118 | 0.000 |  |
|  | NFKB1 rs28362491 | -0.162 | 0.081 | 0.046 |  | NFKB1 rs28362491 | -0.294 | 0.109 | 0.007 |  |
|  | Sex | -0.240 | 0.063 | 0.000 |  | Sex | -0.394 | 0.084 | 0.000 |  |
|  | CCI | 0.067 | 0.015 | 0.000 |  | CCI | 0.104 | 0.020 | 0.000 |  |
|  |  | Estimate | SE | p |  |  | Estimate | SE | p |  |
|  | (Intercept) | 3.250 | 0.092 | 0.000 |  | (Intercept) | 3.486 | 0.123 | 0.000 |  |
|  | NLRP3 rs10754555 | 0.003 | 0.087 | 0.975 |  | NLRP3 rs10754555 | -0.041 | 0.116 | 0.727 |  |
|  | Sex | -0.167 | 0.063 | 0.008 |  | Sex | -0.304 | 0.084 | 0.000 |  |
|  | CCI | 0.062 | 0.015 | 0.000 |  | CCI | 0.099 | 0.019 | 0.000 |  |
|  |  | Estimate | SE | p |  |  | Estimate | SE | p |  |
|  | (Intercept) | 3.481 | 0.233 | 0.000 |  | (Intercept) | 3.676 | 0.317 | 0.000 |  |
|  | PEAR1 rs12041331 | -0.259 | 0.231 | 0.262 |  | PEAR1 rs12041331 | -0.217 | 0.314 | 0.489 |  |
|  | Sex | -0.232 | 0.061 | 0.000 |  | Sex | -0.391 | 0.083 | 0.000 |  |
|  | CCI | 0.068 | 0.014 | 0.000 |  | CCI | 0.103 | 0.019 | 0.000 |  |
|  |  | Estimate | SE | p |  |  | Estimate | SE | p |  |
|  | (Intercept) | 3.172 | 0.088 | 0.000 |  | (Intercept) | 3.447 | 0.119 | 0.000 |  |
|  | rs10108210 | 0.063 | 0.082 | 0.445 |  | rs10108210 | 0.008 | 0.111 | 0.946 |  |
|  | Sex | -0.223 | 0.062 | 0.000 |  | Sex | -0.373 | 0.084 | 0.000 |  |
|  | CCI | 0.069 | 0.014 | 0.000 |  | CCI | 0.105 | 0.019 | 0.000 |  |
|  |  | Estimate | SE | p |  |  | Estimate | SE | p |  |
|  | (Intercept) | 3.259 | 0.080 | 0.000 |  | (Intercept) | 3.507 | 0.109 | 0.000 |  |
|  | rs703297 | -0.058 | 0.073 | 0.428 |  | rs703297 | -0.073 | 0.099 | 0.463 |  |
|  | Sex | -0.214 | 0.062 | 0.001 |  | Sex | -0.377 | 0.084 | 0.000 |  |
|  | CCI | 0.069 | 0.014 | 0.000 |  | CCI | 0.107 | 0.020 | 0.000 |  |
|  |  | Estimate | SE | p |  |  | Estimate | SE | p |  |
|  | (Intercept) | 3.513 | 0.276 | 0.000 |  | (Intercept) | 3.586 | 0.375 | 0.000 |  |
|  | rs713400 | -0.302 | 0.273 | 0.269 |  | rs713400 | -0.138 | 0.372 | 0.711 |  |
|  | Sex | -0.216 | 0.061 | 0.000 |  | Sex | -0.379 | 0.084 | 0.000 |  |
|  | CCI | 0.069 | 0.014 | 0.000 |  | CCI | 0.106 | 0.019 | 0.000 |  |
|  |  | Estimate | SE | p |  |  | Estimate | SE | p |  |
|  | (Intercept) | 3.442 | 0.506 | 0.000 |  | (Intercept) | 3.699 | 0.687 | 0.000 |  |
|  | rs8134378 | -0.237 | 0.501 | 0.637 |  | rs8134378 | -0.256 | 0.680 | 0.707 |  |
|  | Sex | -0.207 | 0.062 | 0.001 |  | Sex | -0.372 | 0.084 | 0.000 |  |
|  | CCI | 0.073 | 0.014 | 0.000 |  | CCI | 0.110 | 0.020 | 0.000 |  |
|  |  | Estimate | SE | p |  |  | Estimate | SE | p |  |
|  | (Intercept) | 3.173 | 0.168 | 0.000 |  | (Intercept) | 3.882 | 0.226 | 0.000 |  |
|  | SLCO1B1 rs4149056 | 0.050 | 0.165 | 0.761 |  | SLCO1B1 rs4149056 | -0.447 | 0.222 | 0.045 |  |
|  | Sex | -0.230 | 0.062 | 0.000 |  | Sex | -0.400 | 0.083 | 0.000 |  |
|  | CCI | 0.069 | 0.014 | 0.000 |  | CCI | 0.108 | 0.019 | 0.000 |  |
|  |  | Estimate | SE | p |  |  | Estimate | SE | p |  |
|  | (Intercept) | 3.187 | 0.087 | 0.000 |  | (Intercept) | 3.413 | 0.118 | 0.000 |  |
|  | TLR1 rs5743551 | 0.053 | 0.082 | 0.519 |  | TLR1 rs5743551 | 0.035 | 0.110 | 0.750 |  |
|  | Sex | -0.216 | 0.062 | 0.001 |  | Sex | -0.363 | 0.084 | 0.000 |  |
|  | CCI | 0.066 | 0.015 | 0.000 |  | CCI | 0.106 | 0.020 | 0.000 |  |
|  |  | Estimate | SE | p |  |  | Estimate | SE | p |  |
|  | (Intercept) | 3.299 | 0.098 | 0.000 |  | (Intercept) | 3.482 | 0.133 | 0.000 |  |
|  | TLR2 rs11938228 | 0.138 | 0.090 | 0.126 |  | TLR2 rs11938228 | 0.171 | 0.123 | 0.166 |  |
|  | Sex | -0.129 | 0.067 | 0.054 |  | Sex | -0.261 | 0.091 | 0.004 |  |
|  | CCI | 0.035 | 0.015 | 0.020 |  | CCI | 0.072 | 0.020 | 0.000 |  |
|  |  | Estimate | SE | p |  |  | Estimate | SE | p |  |
|  | (Intercept) | 3.195 | 0.249 | 0.000 |  | (Intercept) | 3.347 | 0.339 | 0.000 |  |
|  | TLR2 rs1816702 | 0.024 | 0.249 | 0.922 |  | TLR2 rs1816702 | 0.107 | 0.339 | 0.751 |  |
|  | Sex | -0.220 | 0.061 | 0.000 |  | Sex | -0.383 | 0.084 | 0.000 |  |
|  | CCI | 0.070 | 0.014 | 0.000 |  | CCI | 0.107 | 0.020 | 0.000 |  |
|  |  | Estimate | SE | p |  |  | Estimate | SE | p |  |
|  | (Intercept) | 3.173 | 0.096 | 0.000 |  | (Intercept) | 3.354 | 0.130 | 0.000 |  |
|  | TLR2 rs1898830 | 0.058 | 0.089 | 0.519 |  | TLR2 rs1898830 | 0.110 | 0.121 | 0.363 |  |
|  | Sex | -0.203 | 0.063 | 0.001 |  | Sex | -0.357 | 0.085 | 0.000 |  |
|  | CCI | 0.063 | 0.015 | 0.000 |  | CCI | 0.099 | 0.020 | 0.000 |  |
|  |  | Estimate | SE | p |  |  | Estimate | SE | p |  |
|  | (Intercept) | 3.219 | 0.082 | 0.000 |  | (Intercept) | 3.489 | 0.111 | 0.000 |  |
|  | TLR2 rs3804099 | -0.005 | 0.076 | 0.947 |  | TLR2 rs3804099 | -0.048 | 0.103 | 0.645 |  |
|  | Sex | -0.215 | 0.062 | 0.001 |  | Sex | -0.388 | 0.084 | 0.000 |  |
|  | CCI | 0.069 | 0.014 | 0.000 |  | CCI | 0.107 | 0.020 | 0.000 |  |
|  |  | Estimate | SE | p |  |  | Estimate | SE | p |  |
|  | (Intercept) | 3.300 | 0.110 | 0.000 |  | (Intercept) | 3.557 | 0.145 | 0.000 |  |
|  | TLR2 rs7656411 | -0.071 | 0.103 | 0.492 |  | TLR2 rs7656411 | -0.149 | 0.135 | 0.272 |  |
|  | Sex | -0.158 | 0.064 | 0.014 |  | Sex | -0.293 | 0.084 | 0.001 |  |
|  | CCI | 0.061 | 0.015 | 0.000 |  | CCI | 0.099 | 0.019 | 0.000 |  |
|  |  | Estimate | SE | p |  |  | Estimate | SE | p |  |
|  | (Intercept) | 3.259 | 0.117 | 0.000 |  | (Intercept) | 3.451 | 0.159 | 0.000 |  |
|  | TLR4 rs1927911 | -0.056 | 0.112 | 0.621 |  | TLR4 rs1927911 | -0.008 | 0.152 | 0.958 |  |
|  | Sex | -0.215 | 0.062 | 0.001 |  | Sex | -0.378 | 0.084 | 0.000 |  |
|  | CCI | 0.073 | 0.014 | 0.000 |  | CCI | 0.111 | 0.020 | 0.000 |  |
|  |  | Estimate | SE | p |  |  | Estimate | SE | p |  |
|  | (Intercept) | 3.194 | 0.118 | 0.000 |  | (Intercept) | 3.567 | 0.160 | 0.000 |  |
|  | TLR4 rs5030728 | 0.025 | 0.115 | 0.828 |  | TLR4 rs5030728 | -0.127 | 0.155 | 0.412 |  |
|  | Sex | -0.220 | 0.062 | 0.000 |  | Sex | -0.384 | 0.083 | 0.000 |  |
|  | CCI | 0.070 | 0.014 | 0.000 |  | CCI | 0.107 | 0.019 | 0.000 |  |
|  |  | Estimate | SE | p |  |  | Estimate | SE | p |  |
|  | (Intercept) | 3.324 | 0.091 | 0.000 |  | (Intercept) | 3.513 | 0.123 | 0.000 |  |
|  | TLR9 rs187084 | -0.108 | 0.082 | 0.191 |  | TLR9 rs187084 | -0.050 | 0.111 | 0.655 |  |
|  | Sex | -0.212 | 0.062 | 0.001 |  | Sex | -0.375 | 0.084 | 0.000 |  |
|  | CCI | 0.063 | 0.014 | 0.000 |  | CCI | 0.097 | 0.020 | 0.000 |  |
|  |  | Estimate | SE | p |  |  | Estimate | SE | p |  |
|  | (Intercept) | 3.244 | 0.079 | 0.000 |  | (Intercept) | 3.464 | 0.107 | 0.000 |  |
|  | TLR9 rs352162 | -0.039 | 0.073 | 0.594 |  | TLR9 rs352162 | -0.015 | 0.099 | 0.881 |  |
|  | Sex | -0.218 | 0.062 | 0.000 |  | Sex | -0.388 | 0.084 | 0.000 |  |
|  | CCI | 0.070 | 0.014 | 0.000 |  | CCI | 0.107 | 0.019 | 0.000 |  |
|  |  | Estimate | SE | p |  |  | Estimate | SE | p |  |
|  | (Intercept) | 3.115 | 0.177 | 0.000 |  | (Intercept) | 3.289 | 0.240 | 0.000 |  |
|  | TMPRSS2 rs12329760 | 0.109 | 0.174 | 0.529 |  | TMPRSS2 rs12329760 | 0.172 | 0.235 | 0.464 |  |
|  | Sex | -0.223 | 0.062 | 0.000 |  | Sex | -0.391 | 0.084 | 0.000 |  |
|  | CCI | 0.068 | 0.014 | 0.000 |  | CCI | 0.105 | 0.019 | 0.000 |  |
|  |  | Estimate | SE | p |  |  | Estimate | SE | p |  |
|  | (Intercept) | 3.178 | 0.081 | 0.000 |  | (Intercept) | 3.381 | 0.111 | 0.000 |  |
|  | TMPRSS2 rs2070788 | 0.059 | 0.074 | 0.425 |  | TMPRSS2 rs2070788 | 0.103 | 0.101 | 0.307 |  |
|  | Sex | -0.222 | 0.061 | 0.000 |  | Sex | -0.393 | 0.084 | 0.000 |  |
|  | CCI | 0.070 | 0.014 | 0.000 |  | CCI | 0.108 | 0.019 | 0.000 |  |
|  |  | Estimate | SE | p |  |  | Estimate | SE | p |  |
|  | (Intercept) | 3.169 | 0.093 | 0.000 |  | (Intercept) | 3.405 | 0.127 | 0.000 |  |
|  | TMPRSS2 rs463727 | 0.040 | 0.086 | 0.644 |  | TMPRSS2 rs463727 | 0.045 | 0.118 | 0.703 |  |
|  | Sex | -0.217 | 0.062 | 0.001 |  | Sex | -0.387 | 0.085 | 0.000 |  |
|  | CCI | 0.075 | 0.015 | 0.000 |  | CCI | 0.115 | 0.020 | 0.000 |  |
|  |  | Estimate | SE | p |  |  | Estimate | SE | p |  |
|  | (Intercept) | 3.181 | 0.086 | 0.000 |  | (Intercept) | 3.363 | 0.117 | 0.000 |  |
|  | TMPRSS2 rs464397 | 0.030 | 0.080 | 0.707 |  | TMPRSS2 rs464397 | 0.098 | 0.109 | 0.371 |  |
|  | Sex | -0.209 | 0.062 | 0.001 |  | Sex | -0.372 | 0.084 | 0.000 |  |
|  | CCI | 0.073 | 0.014 | 0.000 |  | CCI | 0.110 | 0.019 | 0.000 |  |
|  |  | Estimate | SE | p |  |  | Estimate | SE | p |  |
|  | (Intercept) | 3.319 | 0.072 | 0.000 |  | (Intercept) | 3.527 | 0.098 | 0.000 |  |
|  | TMPRSS2 rs55964536 | -0.122 | 0.067 | 0.071 |  | TMPRSS2 rs55964536 | -0.076 | 0.092 | 0.409 |  |
|  | Sex | -0.240 | 0.065 | 0.000 |  | Sex | -0.416 | 0.088 | 0.000 |  |
|  | CCI | 0.071 | 0.015 | 0.000 |  | CCI | 0.109 | 0.020 | 0.000 |  |
|  |  | Estimate | SE | p |  |  | Estimate | SE | p |  |
|  | (Intercept) | 3.181 | 0.082 | 0.000 |  | (Intercept) | 3.423 | 0.112 | 0.000 |  |
|  | TMPRSS2 rs734056 | 0.029 | 0.075 | 0.704 |  | TMPRSS2 rs734056 | 0.024 | 0.102 | 0.813 |  |
|  | Sex | -0.211 | 0.062 | 0.001 |  | Sex | -0.376 | 0.084 | 0.000 |  |
|  | CCI | 0.073 | 0.014 | 0.000 |  | CCI | 0.111 | 0.019 | 0.000 |  |
|  |  | Estimate | SE | p |  |  | Estimate | SE | p |  |
|  | (Intercept) | 2.992 | 0.082 | 0.000 |  | (Intercept) | 3.227 | 0.109 | 0.000 |  |
|  | TMPRSS2 rs75603675 | 0.284 | 0.072 | 0.000 |  | TMPRSS2 rs75603675 | 0.226 | 0.096 | 0.019 |  |
|  | Sex | -0.211 | 0.064 | 0.001 |  | Sex | -0.361 | 0.085 | 0.000 |  |
|  | CCI | 0.070 | 0.015 | 0.000 |  | CCI | 0.107 | 0.020 | 0.000 |  |
|  |  | Estimate | SE | p |  |  | Estimate | SE | p |  |
|  | (Intercept) | 3.151 | 0.307 | 0.000 |  | (Intercept) | 3.491 | 0.416 | 0.000 |  |
|  | TMPRSS2 rs77675406 | 0.065 | 0.305 | 0.832 |  | TMPRSS2 rs77675406 | -0.041 | 0.413 | 0.922 |  |
|  | Sex | -0.221 | 0.062 | 0.000 |  | Sex | -0.394 | 0.084 | 0.000 |  |
|  | CCI | 0.069 | 0.014 | 0.000 |  | CCI | 0.107 | 0.019 | 0.000 |  |
|  |  | Estimate | SE | p |  |  | Estimate | SE | p |  |
|  | (Intercept) | 3.150 | 0.138 | 0.000 |  | (Intercept) | 3.345 | 0.187 | 0.000 |  |
|  | TNF rs1799964 | 0.075 | 0.134 | 0.578 |  | TNF rs1799964 | 0.117 | 0.182 | 0.522 |  |
|  | Sex | -0.226 | 0.061 | 0.000 |  | Sex | -0.392 | 0.083 | 0.000 |  |
|  | CCI | 0.067 | 0.014 | 0.000 |  | CCI | 0.104 | 0.019 | 0.000 |  |
|  |  | Estimate | SE | p |  |  | Estimate | SE | p |  |
|  | (Intercept) | 3.239 | 0.159 | 0.000 |  | (Intercept) | 3.497 | 0.215 | 0.000 |  |
|  | TNF rs1800610 | -0.028 | 0.154 | 0.853 |  | TNF rs1800610 | -0.052 | 0.208 | 0.803 |  |
|  | Sex | -0.211 | 0.062 | 0.001 |  | Sex | -0.373 | 0.084 | 0.000 |  |
|  | CCI | 0.072 | 0.014 | 0.000 |  | CCI | 0.109 | 0.020 | 0.000 |  |
|  |  | Estimate | SE | p |  |  | Estimate | SE | p |  |
|  | (Intercept) | 2.933 | 0.276 | 0.000 |  | (Intercept) | 3.325 | 0.374 | 0.000 |  |
|  | TNF rs1800629 | 0.277 | 0.275 | 0.313 |  | TNF rs1800629 | 0.125 | 0.373 | 0.737 |  |
|  | Sex | -0.215 | 0.062 | 0.001 |  | Sex | -0.386 | 0.084 | 0.000 |  |
|  | CCI | 0.073 | 0.014 | 0.000 |  | CCI | 0.109 | 0.019 | 0.000 |  |
|  |  | Estimate | SE | p |  |  | Estimate | SE | p |  |
|  | (Intercept) | 2.839 | 0.354 | 0.000 |  | (Intercept) | 2.876 | 0.479 | 0.000 |  |
|  | TNF rs361525 | 0.366 | 0.354 | 0.301 |  | TNF rs361525 | 0.570 | 0.479 | 0.234 |  |
|  | Sex | -0.209 | 0.062 | 0.001 |  | Sex | -0.382 | 0.083 | 0.000 |  |
|  | CCI | 0.074 | 0.014 | 0.000 |  | CCI | 0.111 | 0.019 | 0.000 |  |
|  |  | Estimate | SE | p |  |  | Estimate | SE | p |  |
|  | (Intercept) | 2.985 | 0.127 | 0.000 |  | (Intercept) | 3.261 | 0.172 | 0.000 |  |
|  | TNKA rs1800630 | 0.245 | 0.122 | 0.045 |  | TNKA rs1800630 | 0.227 | 0.165 | 0.169 |  |
|  | Sex | -0.211 | 0.064 | 0.001 |  | Sex | -0.380 | 0.087 | 0.000 |  |
|  | CCI | 0.070 | 0.015 | 0.000 |  | CCI | 0.098 | 0.020 | 0.000 |  |
|  |  | Estimate | SE | p |  |  | Estimate | SE | p |  |
|  | (Intercept) | 3.266 | 0.390 | 0.000 |  | (Intercept) | 3.228 | 0.526 | 0.000 |  |
|  | TRAF3IP2 rs13190932 | -0.059 | 0.387 | 0.878 |  | TRAF3IP2 rs13190932 | 0.219 | 0.523 | 0.676 |  |
|  | Sex | -0.214 | 0.062 | 0.001 |  | Sex | -0.372 | 0.083 | 0.000 |  |
|  | CCI | 0.073 | 0.014 | 0.000 |  | CCI | 0.107 | 0.019 | 0.000 |  |
|  |  | Estimate | SE | p |  |  | Estimate | SE | p |  |
|  | (Intercept) | 3.389 | 0.357 | 0.000 |  | (Intercept) | 3.361 | 0.483 | 0.000 |  |
|  | TRAF3IP2 rs13196377 | -0.185 | 0.354 | 0.601 |  | TRAF3IP2 rs13196377 | 0.082 | 0.479 | 0.865 |  |
|  | Sex | -0.212 | 0.062 | 0.001 |  | Sex | -0.384 | 0.084 | 0.000 |  |
|  | CCI | 0.072 | 0.014 | 0.000 |  | CCI | 0.110 | 0.019 | 0.000 |  |
|  |  | Estimate | SE | p |  |  | Estimate | SE | p |  |
|  | (Intercept) | 3.261 | 0.390 | 0.000 |  | (Intercept) | 3.214 | 0.529 | 0.000 |  |
|  | TRAF3IP2 rs33980500 | -0.062 | 0.388 | 0.874 |  | TRAF3IP2 rs33980500 | 0.224 | 0.526 | 0.671 |  |
|  | Sex | -0.205 | 0.062 | 0.001 |  | Sex | -0.370 | 0.084 | 0.000 |  |
|  | CCI | 0.074 | 0.014 | 0.000 |  | CCI | 0.111 | 0.019 | 0.000 |  |
|  |  | Estimate | SE | p |  |  | Estimate | SE | p |  |
|  | (Intercept) | 3.183 | 0.089 | 0.000 |  | (Intercept) | 3.340 | 0.121 | 0.000 |  |
|  | VDR rs2228570 | 0.031 | 0.083 | 0.710 |  | VDR rs2228570 | 0.126 | 0.113 | 0.263 |  |
|  | Sex | -0.209 | 0.062 | 0.001 |  | Sex | -0.374 | 0.084 | 0.000 |  |
|  | CCI | 0.073 | 0.014 | 0.000 |  | CCI | 0.110 | 0.019 | 0.000 |  |
|  |  | Estimate | SE | p |  |  | Estimate | SE | p |  |
|  | (Intercept) | 3.122 | 0.077 | 0.000 |  | (Intercept) | 3.333 | 0.104 | 0.000 |  |
|  | ABCB1 rs1045642 | 0.099 | 0.071 | 0.165 |  | ABCB1 rs1045642 | 0.132 | 0.096 | 0.170 |  |
|  | Sex | -0.204 | 0.062 | 0.001 |  | Sex | -0.373 | 0.084 | 0.000 |  |
|  | CCI | 0.073 | 0.014 | 0.000 |  | CCI | 0.111 | 0.019 | 0.000 |  |
|  |  | Estimate | SE | p |  |  | Estimate | SE | p |  |
|  | (Intercept) | 3.175 | 0.083 | 0.000 |  | (Intercept) | 3.387 | 0.113 | 0.000 |  |
|  | ABCB1 rs1128503 | 0.040 | 0.079 | 0.613 |  | ABCB1 rs1128503 | 0.073 | 0.106 | 0.490 |  |
|  | Sex | -0.214 | 0.062 | 0.001 |  | Sex | -0.386 | 0.084 | 0.000 |  |
|  | CCI | 0.072 | 0.014 | 0.000 |  | CCI | 0.109 | 0.019 | 0.000 |  |
|  |  | Estimate | SE | p |  |  | Estimate | SE | p |  |
|  | (Intercept) | 3.170 | 0.057 | 0.000 |  | (Intercept) | 3.382 | 0.079 | 0.000 |  |
|  | Obesity | 0.278 | 0.084 | 0.001 |  | Obesity | 0.358 | 0.116 | 0.002 |  |
|  | Sex | -0.186 | 0.062 | 0.003 |  | Sex | -0.352 | 0.085 | 0.000 |  |
|  | CCI | 0.068 | 0.014 | 0.000 |  | CCI | 0.117 | 0.019 | 0.000 |  |
|  |  | Estimate | SE | p |  |  | Estimate | SE | p |  |
|  | (Intercept) | 3.162 | 0.057 | 0.000 |  | (Intercept) | 3.377 | 0.078 | 0.000 |  |
|  | Dyslipidemia | 0.302 | 0.068 | 0.000 |  | Dyslipidemia | 0.356 | 0.093 | 0.000 |  |
|  | Sex | -0.200 | 0.061 | 0.001 |  | Sex | -0.369 | 0.084 | 0.000 |  |
|  | CCI | 0.051 | 0.015 | 0.001 |  | CCI | 0.099 | 0.020 | 0.000 |  |
|  |  | Estimate | SE | p |  |  | Estimate | SE | p |  |
|  | (Intercept) | 3.194 | 0.059 | 0.000 |  | (Intercept) | 3.403 | 0.081 | 0.000 |  |
|  | Tobacco | 0.065 | 0.072 | 0.367 |  | Tobacco | 0.124 | 0.099 | 0.208 |  |
|  | Sex | -0.190 | 0.063 | 0.003 |  | Sex | -0.350 | 0.086 | 0.000 |  |
|  | CCI | 0.070 | 0.014 | 0.000 |  | CCI | 0.118 | 0.020 | 0.000 |  |
|  |  | Estimate | SE | p |  |  | Estimate | SE | p |  |
|  | (Intercept) | 3.203 | 0.057 | 0.000 |  | (Intercept) | 3.428 | 0.078 | 0.000 |  |
|  | Alcohol | 0.085 | 0.109 | 0.437 |  | Alcohol | 0.072 | 0.149 | 0.628 |  |
|  | Sex | -0.193 | 0.063 | 0.002 |  | Sex | -0.364 | 0.086 | 0.000 |  |
|  | CCI | 0.070 | 0.014 | 0.000 |  | CCI | 0.121 | 0.020 | 0.000 |  |
|  |  | Estimate | SE | p |  |  | Estimate | SE | p |  |
|  | (Intercept) | 3.189 | 0.056 | 0.000 |  | (Intercept) | 3.413 | 0.078 | 0.000 |  |
|  | AECIs | 0.312 | 0.091 | 0.001 |  | AECIs | 0.307 | 0.126 | 0.015 |  |
|  | Sex | -0.196 | 0.061 | 0.001 |  | Sex | -0.366 | 0.085 | 0.000 |  |
|  | CCI | 0.063 | 0.014 | 0.000 |  | CCI | 0.114 | 0.020 | 0.000 |  |
|  |  | Estimate | SE | p |  |  | Estimate | SE | p |  |
|  | (Intercept) | 3.200 | 0.056 | 0.000 |  | (Intercept) | 3.417 | 0.077 | 0.000 |  |
|  | ARA-II | 0.197 | 0.088 | 0.026 |  | ARA-II | 0.311 | 0.121 | 0.010 |  |
|  | Sex | -0.196 | 0.062 | 0.002 |  | Sex | -0.364 | 0.085 | 0.000 |  |
|  | CCI | 0.063 | 0.015 | 0.000 |  | CCI | 0.109 | 0.020 | 0.000 |  |
|  |  | Estimate | SE | p |  |  | Estimate | SE | p |  |
|  | (Intercept) | 3.212 | 0.056 | 0.000 |  | (Intercept) | 3.434 | 0.078 | 0.000 |  |
|  | Antialdosterone drugs | 0.363 | 0.313 | 0.245 |  | Antialdosterone drugs | 0.175 | 0.430 | 0.683 |  |
|  | Sex | -0.201 | 0.062 | 0.001 |  | Sex | -0.371 | 0.085 | 0.000 |  |
|  | CCI | 0.070 | 0.014 | 0.000 |  | CCI | 0.122 | 0.020 | 0.000 |  |
|  |  | Estimate | SE | p |  |  | Estimate | SE | p |  |
|  | (Intercept) | 3.212 | 0.056 | 0.000 |  | (Intercept) | 3.436 | 0.078 | 0.000 |  |
|  | Anticoagulant drugs | 0.183 | 0.163 | 0.260 |  | Anticoagulant drugs | 0.255 | 0.223 | 0.254 |  |
|  | Sex | -0.199 | 0.062 | 0.001 |  | Sex | -0.368 | 0.085 | 0.000 |  |
|  | CCI | 0.068 | 0.015 | 0.000 |  | CCI | 0.117 | 0.020 | 0.000 |  |
|  |  | Estimate | SE | p |  |  | Estimate | SE | p |  |
|  | (Intercept) | 3.210 | 0.056 | 0.000 |  | (Intercept) | 3.434 | 0.077 | 0.000 |  |
|  | Corticosteroids | 0.357 | 0.190 | 0.060 |  | Corticosteroids | 0.343 | 0.261 | 0.189 |  |
|  | Sex | -0.206 | 0.062 | 0.001 |  | Sex | -0.376 | 0.085 | 0.000 |  |
|  | CCI | 0.069 | 0.014 | 0.000 |  | CCI | 0.120 | 0.020 | 0.000 |  |
|  |  | Estimate | SE | p |  |  | Estimate | SE | p |  |
|  | (Intercept) | 3.212 | 0.057 | 0.000 |  | (Intercept) | 3.436 | 0.078 | 0.000 |  |
|  | Immunosuppressants | -0.080 | 0.193 | 0.680 |  | Immunosuppressants | -0.129 | 0.265 | 0.627 |  |
|  | Sex | -0.201 | 0.062 | 0.001 |  | Sex | -0.371 | 0.085 | 0.000 |  |
|  | CCI | 0.072 | 0.014 | 0.000 |  | CCI | 0.123 | 0.020 | 0.000 |  |
|  |  | Estimate | SE | p |  |  | Estimate | SE | p |  |
|  | (Intercept) | 2.884 | 0.086 | 0.000 |  | (Intercept) | 3.120 | 0.106 | 0.000 |  |
|  | Biogeographic group | 0.157 | 0.108 | 0.335 |  | Biogeographic group | 0.128 | 0.133 | 0.335 |  |
|  | Sex | -0.308 | 0.089 | 0.001 |  | Sex | -0.483 | 0.110 | 0.000 |  |
|  | CCI | 0.104 | 0.021 | 0.000 |  | CCI | 0.155 | 0.025 | 0.000 |  |

WHOCS-1: Modified World Health Organization Covid-19 severity scale at first hospital examination. WHOCS-2: Highest score on the modified World Health Organisation Covid-19 severity scale; CCI: Charlson comorbidity index; ACEIs: angiotensin converting enzyme inhibitors; ARA-II: angiotensin recepetor II antagonists; SE: standard error. *p*: nominal significance; *p*’: significance after Bonferroni correction for multiple comparisons.
